# Supplementary material for: Quartz-Seq2: a high-throughput single-cell RNA-sequencing method that effectively uses limited sequence reads
Source: Genome Biol. 2018 Mar 9;19:29. doi: 10.1186/s13059-018-1407-3 (PMC5845169; doi:10.1186/s13059-018-1407-3)
Supplement: Supplementary file 1 — Supplemental note and supplemental figures. (PDF 12818 kb) [file 13059_2018_1407_MOESM1_ESM.pdf]

Supplemental Note and Supplemental Figures

Quartz-Seq2: a high-throughput single-cell RNA-sequencing method that effectively uses limited sequence reads

Yohei Sasagawa[1†], Hiroki Danno[1†], Hitomi Takada[2†], Masashi Ebisawa[1], Kaori Tanaka[1], Tetsutaro Hayashi[1], Akira Kurisaki[2*], and Itoshi Nikaido[1,3*]

[†]These authors contributed equally to this work.

*Corresponding author

[1]Bioinformatics Research Unit, Advanced Center for Computing and Communication, RIKEN, Hirosawa 2-1, Wako, Saitama, Japan

[2]Laboratory of Stem Cell Technology, Graduate School of Biological Sciences, Nara Institute of Science and Technology, Takayama-cho 8916-5, Ikoma, Nara, Japan

[3]Single-cell Omics Research Unit, RIKEN Center for Developmental Biology. 2-2-3 Minatojima-minamimachi, Chuo-ku, Kobe, Japan

Supplemental Note

RT primer and byproduct synthesis

We reported in our previous Quartz-Seq paper that the poly-A tagging strategy has great potential for increasing the number of genes detected [1]. However, the efficiency of poly-A tagging itself for single-cell RNA-seq has not been improved. To convert more cDNA molecules to amplifiable cDNA, we attempted to improve the poly-A tagging efficiency (Additional file 1: Fig. S1). However, we should first focus on suppressing the synthesis of byproducts because the byproducts from whole-transcript amplification cause severe problems that prevent quantitative performance via a low mapping ratio [1].

It has been reported that byproducts could be synthesized from reverse-transcription (RT) primers in poly-A tagging-based methods ([1-3], Additional file 1: Fig. S2a). Quartz-Seq required RT primer digestion by exonuclease I treatment to suppress byproduct synthesis. We thus investigated whether column purification could be used instead of exonuclease I treatment. The cDNA purified using columns was used for whole-transcript amplification based on conditions similar to those in Quartz-Seq. We found that cDNA purified with original Quartz-Seq RT primer (70-mer) was not associated with the synthesis of byproducts upon column purification for the pooling of cell-barcoded cDNA. On the other hand, we observed an enormous amount of byproduct synthesis when using long RT primer (126-mer). The length of DNA thus affects the tendency for byproduct synthesis [1]. Therefore, we designed a relatively short RT primer (v3.1: 73-mer), which has a cell-barcode sequence and a UMI sequence. This is designed based on the RT primer conjugated to barcoded beads of Drop-seq. This is because Drop-seq RT primer is relatively short, at the same length as the original Quartz-seq primer [1,4]. By using the v3.1 RT primer, we succeeded in amplifying cDNA without byproduct synthesis (Additional file 1: Fig. S2b). Thereafter, we used v3.1 RT primers for the technical development of Quartz-Seq2.

Streamline for pooling of cell-barcoded cDNA

After cell barcoding in reverse transcription, labeled cDNA is pooled and purified for subsequent whole-transcript amplification. A low volume of reverse-transcription solution for cell barcoding is efficient for cost reduction [4-6]. On the other hand, the pooling of labeled cDNA at a low volume from a PCR plate is complicated and time-consuming using pipette tips. Ideally, cell-barcoded cDNA should be pooled and purified with minimal loss for the subsequent whole-transcript amplification step in an expeditious manner. Therefore, we validated and streamlined the pooling step of cell-barcoded cDNA at a low volume. We collected 2 μL of solution from every well of a 384-well PCR plate and estimated the collection efficiency. By using an eight-channel pipette, we collected 79.6±2.4% (n=3) of solution in approximately 10 min from every well of a 384-well PCR plate. We also developed a spin-down collection system for the PCR plate (Figure 1 and Additional file 1: Fig. S4). Using this collection system (type A), we collected 88.7±0.99% (n=3) of solution in 5 min from two to four plates. The collection efficiency of cDNA solution was improved by approximately 10% by constructing this spin-down collection system. In addition, the collection time per 384-well plate using this collection system was four- to eightfold faster than with a multi-channel pipette. We also validated the purification efficiency of cell-barcoded cDNA. We determined the qPCR score with eight genes using purified first-strand cDNA and unpurified first-strand cDNA. The purification efficiency was 93.77% (n=4) (Additional file 1: Fig S3a). By combining our approach with the spin-down collection system, we estimated that 80%–82% of cell-barcoded cDNA could theoretically be used for subsequent whole-transcript amplification in our system.

To import more cDNA molecules into subsequent whole-transcript amplification, we attempted to improve the efficiency of conversion from target RNA to first-strand cDNA in reverse transcription. First, we performed reverse transcription under conditions with various buffers and temperatures (Additional file 1: Fig. S3b). The Spearman's rank correlation coefficient (SCC) between the Quartz-Seq-like condition (PCR buffer, 45ºC, 20 min) and positive control was 0.6957. In contrast, the SCC between the modified condition (T100, 50ºC, 50 min) and the positive control was 0.8996. Moreover, reverse-transcription efficiency was slightly improved by approximately 20% on average (Additional file 1: Fig. S3b). These results show that

the modified RT condition reduced the bias of sequence preference in reverse transcription. In addition, we could import more cDNA molecules into subsequent whole-transcript amplification.

Relationship between sequence platform and Quartz-Seq2

We also focused on the relationship between sequencing platform and Quartz-Seq2. We did not observe clear differences between the NextSeq500 and HiSeq2500 sequencing platforms for determination of the UMI count and the gene count for Quartz-Seq2 (Additional file 1: Fig. S14a). On the other hand, the unique mapping ratio and UMI count clearly depended on the Read2 length for transcript mapping (Additional file 1: Fig. S14c, d). In this regard, the number of detected genes slightly depended on Read2 length (Additional file 1: Fig. S14d). Long Read2 length led to better UMI counts, but high sequencing cost (Additional file 1: Fig. S14b). Therefore, in this study, we mainly used the NextSeq500 sequence platform and a Read2 length of 62 due to the cost performance of this combination.

Library preparation method for Quartz-Seq2

Moreover, we mainly used the adaptor ligation method for sequence library preparation of Quartz-Seq2. We did not observe a clear difference between the two sequence library preparation methods (ligation and Nextera/Tn5 transposase) regarding determination of the UMI count and the gene count. However, we could easily identify specific familiar genes (such as *Pou5f1* and *Gapdh*) using the ligation method (Additional file 1: Fig. S14e). Therefore, we mainly used the ligation method for Quartz-Seq2.

References

1. Sasagawa Y, Nikaido I, Hayashi T, Danno H, Uno KD, Imai T, et al. Quartz-Seq: a highly reproducible and sensitive single-cell RNA sequencing method, reveals non-genetic gene-expression heterogeneity. Genome Biol. 2013;14:R31.

2. Tang F, Barbacioru C, Wang Y, Nordman E, Lee C, Xu N, et al. mRNA-Seq whole-transcriptome analysis of a single cell. Nat. Methods. 2009;6:377–82.
3. Kurimoto K. An improved single-cell cDNA amplification method for efficient high-density oligonucleotide microarray analysis. Nucleic Acids Res. 2006;34:e42–2.
4. Macosko EZ, Basu A, Satija R, Nemesh J, Shekhar K, Goldman M, et al. Highly Parallel Genome-wide Expression Profiling of Individual Cells Using Nanoliter Droplets. Cell. 2015;161:1202–14.
5. Klein AM, Mazutis L, Akartuna I, Tallapragada N, Veres A, Li V, et al. Droplet Barcoding for Single-Cell Transcriptomics Applied to Embryonic Stem Cells. Cell. 2015;161:1187–201.
6. Bose S, Wan Z, Carr A, Rizvi A, Vieira G, Pe’er D, et al. Scalable microfluidics for single-cell RNA printing and sequencing. Genome Biol. 2015;16:120.

Supplemental Figures

Fig. S1. Summary of improvements conferred by Quartz-Seq2

Target RNA was converted into amplifiable cDNA with a cell barcode sequence via several steps. We show a summary of the improvements in each step in the boxes. To convert more mRNA molecules to first-strand cDNA, we improved the efficiency of reverse transcription. Moreover, we used low-enzyme concentration for reverse-transcription. To collect more cDNA molecules for subsequent steps, we improved the collection efficiency in pooling steps. In addition, to convert more cDNA molecules to amplifiable cDNA, we improved the poly-A tagging efficiency. Finally, we reduced the PCR bias using an UMI sequence in the reverse-transcription primer.

Fig. S2. Suppression of byproduct synthesis for poly-A tagging strategy

a) A schematic representation of the suppression of byproduct synthesis. RT primer remained in purified cDNA solution. Survival RT primer could be targeted with terminal transferase to attach a poly-A tail, the same as in the first-strand cDNA. Both types of resulting DNA had complementary sequences at both ends. The total length of the byproduct was shorter than first-strand cDNA. Previously, we reported that the length of target DNA is important for the suppression of byproduct synthesis. Specifically, the byproduct tended to form a pan-like structure, which prevented binding between PCR primer and template DNA. Finally, byproduct amplification was suppressed.

b) The effect of RT primer on byproduct synthesis. We used three kinds of RT primer (Quartz-Seq RT primer, v2 RT primer, and v3.1 RT primer) for whole-transcript amplification, in accordance with the Quartz-Seq-like conditions. First-strand cDNA with respective RT primer was treated with column purification and/or exonuclease I digestion. After that, purified cDNA solution was used for whole-transcript amplification. The yellow arrowhead indicates the byproduct derived from RT primer.

c) Typical cDNA pattern of Quartz-Seq2 from a 384-well plate containing 10 pg of total RNA.

Fig. S3. The efficiency of first-strand cDNA synthesis and column purification

a) We validated the purification efficiency. We prepared first-strand cDNA with 1 ng of total RNA and 1 ng of total RNA with 10 ng of tRNA using oligo-dT primer (mixture of v3.1). We performed column purification for first-strand cDNA solution using DNA Clean & Concentrator™-5 kit. Thereafter, we determined the qPCR scores for eight genes (*Eef1b2*, *Nanog*, *Pou5f1*, *Sox2*, *Utf1*, *Spp1*, *Ywhae*, and *Tbp*) using purified first-strand cDNA and unpurified first-strand cDNA. We estimated the purification efficiency using the slope score of linear regression analysis by comparison between unpurified and purified conditions. The purification efficiency was 93.77% (n=4). The 10 ng of tRNA as carrier RNA. We thus investigated whether column purification could be used instead of exonuclease I treatment, which reached 95.52% (n=4) (p-value, 0.987). SCC stands for Spearman's rank correlation coefficient, while PCC stands for Pearson's correlation coefficient.

b) Improvement of reverse-transcription efficiency. We prepared first-strand cDNA using 200 pg of total RNA and poly-dT primer (mixture of v3.1) under six conditions (n=4). We also prepared first-strand cDNA using 200 pg of total RNA and random hexamer as a control for comparison (n=4). For the reverse transcription with random hexamer, we used the SuperScript IV system in accordance with the manufacturer's instructions. We determined qPCR scores for eight genes (*Eef1b2*, *Nanog*, *Pou5f1*, *Sox2*, *Utf1*, *Spp1*, *Ywhae*, and *Tbp*) using the above cDNA. We describe the respective conditions (buffers and temperature conditions) for reverse transcription with poly-dT primer in the boxes. In Quartz-Seq, the reverse-transcription buffer and temperature conditions were as follows (PCR buffer and 35ºC for 5 min, 45ºC for 20 min). Finally, we adopted the following conditions for Quartz-Seq2 (T100 buffer and 35ºC for 5 min, 50ºC for 50 min). Details of the buffer compositions are presented in Additional file 5: Table S4. We estimated the relative efficiency of reverse transcription using the slope score of linear regression analysis by comparison between poly-dT conditions and random hexamer conditions. In the Quartz-Seq condition, the relative efficiency of reverse transcription was 48.48%. In the Quartz-Seq2 condition, the relative efficiency was 59.02%.

Fig. S4. How to assemble collector unit for pooling of cell-barcoded cDNA

a) Dimensional outline of the stainless-steel frame adaptor for collector type A. The metal frame was made by bending and welding one stainless-steel panel at a small factory (Yakkensha or AS ONE Corporation).

b) Assembly of collector type A. (1) First, we prepared a disposable one-well reservoir (Nalgene Microplate Robotic Reservoir with Flat Bottom 1200-1301, Thermo Scientific). (2) We put the metal frame around the one-well reservoir. (3) To prevent the spilling of liquid from the source plate to the reservoir plate, we sealed the foresides of the reservoir with paraffin film (Parafilm). (4) We then turned the reaction 384-well PCR plate upside down on the assembled collector type A. (5) Next, we set it on a centrifuge adaptor. (6) We then centrifuged the plate with the assemble collector at 3,010 g and 4ºC for 3 min with swing-bucket rotors. By using spin-down collection (type A), we collected 88.7±0.99% (n=3) of solution in 5 min from two to four plates. We mainly used type A for cDNA collection.

c) Assembly of collector type B. (0) By using type B, cDNA solution of 384 wells was collected and split into eight wells in a reservoir. (1) We prepared an eight-well disposable reservoir. (2) We put a joint-adaptor (384 Transfer Plate 1859-3845, Watson/Fukaekasei) around the eight-well reservoir. The joint-adaptor has 384 funnel-shaped wells, which enable liquid transfer. (3) We turned the reaction 384-well PCR plate upside down on the assembled collector type B. (4) We then fixed the assembled one using metal holders (Watson/Fukaekasei). (5) Next, we set it on a centrifuge adaptor. (6) We centrifuged the plate with the assemble collector at 3,010 g and 4ºC for 3 min with swing-bucket rotors. By using spin-down collection (type B), we collected 86.9±0.4% (n=2) of solution in 5 min from two to four plates.

Fig. S5. Reproducibility of improvement of poly-A tagging reaction

a) Bar plot represents the relative DNA yield in various poly-A tagging conditions using first-strand cDNA purified from 1 ng of total RNA (n=3 for each condition). We quantified cDNA yield (400–5,900 bp) and byproduct DNA yield (100–400 bp) by Fragment Analyzer (Advanced Analytical). A red color indicates the conditions for which Quartz-Seq2 was used.

b) Another experiment for the improvement of poly-A tagging efficiency with T55 buffer and the "Increment" condition.

c) The improvement of cDNA yield with the combination of T55 buffer and "Increment" (T55+Inc) was confirmed by qPCR assay. We detected eight genes (*Eef1b2*, *Nanog*, *Pou5f1*, *Sox2*, *Utf1*, *Spp1*, *Ywhae*, *Tbp*) with amplified cDNA of Figure 2a and nonamplified cDNA. We prepared nonamplified cDNA with 200 pg of total RNA and random hexamer. SCC stands for Spearman's rank correlation coefficient.

Fig. S6. Cost comparison of single-cell RNA-seq methods including Quartz-Seq2

a) Costs of sequence library DNA and sequence analysis with NextSeq500 for single-cell RNA-seq methods. Quartz-Seq refers to the method that we previously developed. We show the cost in Japanese currency [the monetary unit is the yen (¥)]. The exchange rates for one US dollar and one euro were 113 yen and 131 yen on average in November 2017. The blue bar represents the sequence cost, if we prepare 0.1 M or 0.2 M fastq reads on average for a single cell. The cost of several methods is derived from a previous study (asterisks) [1].

b) The relative total cost including preparation cost and sequence cost for single-cell RNA-seq methods.

1. Ziegenhain C, Vieth B, Parekh S, Reinius B, Guillaumet-Adkins A, Smets M, et al. Comparative Analysis of Single-Cell RNA Sequencing Methods. Mol. Cell. 2017;65:631–4.

Fig. S7. Cost reduction via a low concentration of enzymes in reverse transcription for Quartz-Seq2

a) The step of reverse transcription has the highest cost in the single-cell experimental part of Quartz-Seq2. We present the different proportions of costs associated with each of the experimental steps. The step of reverse transcription accounts for approximately 65% of the total cost in the "RT100" condition. We thus established the "RT25" condition by reducing the enzyme concentration to 25% in reverse transcription (for details, see Methods). Moreover, the number

of columns for the concentration of first-strand cDNA affected the cost for the process downstream of purifying cDNA. The use of a small number of columns also decreased the cost.

b) Assay assessing the use of a low enzyme concentration in reverse transcription. We reverse-transcribed 200 pg of total RNA into first-strand cDNA with two buffers (PCR buffer and T100 buffer). T100 buffer was used for the reverse-transcription step of Quartz-Seq2. We reduced the levels of reverse transcriptase and RNase inhibitor in the reverse-transcription premix as follows: RT100 (20 units/μL SuperScript III, 2.2 units/μL RNasin plus), RT80 (16 units/μL SuperScript III, 1.76 units/μL RNasin plus), RT60 (12 units/μL SuperScript III, 1.32 units/μL RNasin plus), RT40 (8 units/μL SuperScript III, 0.88 units/μL RNasin plus), and RT20 (4 units/μL SuperScript III, 0.44 units/μL RNasin plus). We determined the expression of four genes by qPCR for the respective conditions (n=4). We show the relative qPCR score, which is normalized by RT100 with PCR buffer for the respective genes.

c) We prepared first-strand cDNA using 200 pg of total RNA and poly-dT primer (mixture of v3.1) under serially diluted enzyme conditions (technical replicates, n=5). The detail of enzyme concentration is presented in the figure. We determined qPCR scores for ten genes (*Eef1b2*, *Nanog*, *Pou5f1*, *Sox2*, *Fn1*, *Spp1*, *Utf1*, *Atp5a*, *Rex1*, and *Lefty2*) using the above cDNA. For Figure 2b, we calculated the average relative qPCR score with 10 genes per technical replicate.

d) Bar plots present the amount of amplified cDNA from three technical replicates of a 384-well plate with 10 pg of total RNA in all wells for the "RT100" and "RT25" conditions. The y-axis represents the yield of amplified cDNA. The cDNA yields from 10 pg of total RNA were 55.9±5.5 ng for "RT100" and 65.7±2.5 ng for "RT25." The presented p-value was obtained using two-tailed Welch's t-test.

Fig. S8. Low enzyme concentration in reverse transcription improved the variability of UMI counts and gene counts

For three conditions [Quartz-Seq-like, Quartz-Seq (RT100), Quartz-Seq (RT25)], we performed three batch experiments using all wells of a 384-well plate, which contained 10 pg of total RNA. For Quartz-Seq2, we used different enzyme concentrations for reverse transcription. We used the following reverse-transcription premix: RT100 (2x Thermopol buffer, 20 units/μL SuperScript

III, 2.2 units/μL RNasin plus) and RT25 (2x Thermopol buffer, 5 units/μL SuperScript III, 0.55 units/μL RNasin plus) for Quartz-Seq2. The presented p-value was obtained using two-tailed Welch's t-test between the RT100 condition (three 384-well plates) and the RT25 condition (three 384-well plates). The UMI conversion efficiency levels at approximately 0.096 M fastq reads on average per well were as follows: Quartz-Seq-like (14.83±1.3%, three 384-well plates), Quartz-Seq2 (RT100) (27.76±1.05%, three 384-well plates), and Quartz-Seq2 (RT25) (31.25±0.81%, three 384-well plates). We prepared library DNA with Tn5 transposase for the batch 3 experiment for the Quartz-Seq-like condition (asterisks). For accurate comparison of quantitative performance, we removed batch 3 of Quartz-Seq-like in Figure 2.
b) We analyzed the same sequence library DNA with NextSeq500 and HiSeq2500. Read2 lengths of NextSeq500 and HiSeq2500 were 62 and 98, respectively. Circles represent the data from NextSeq500. Triangles represent the data from HiSeq2500.

Fig. S9. Calculation of UMI conversion efficiency

We performed the conversion from fastq reads as initial reads to UMI counts via several steps. We define the formula for calculating the UMI conversion efficiency (boxed region). Each parameter is defined as follows: UMI_{sc}: the number of UMI count, assigned to a single-cell sample, $fastq_{sc}$: the number of fastq reads derived from each single-cell sample, $fastq_{non\text{-}sc}$: the number of fastq reads derived from non-single-cell samples, which include experimental byproducts such as WTA adaptors, WTA byproducts, and non-STAMPs. Initial fastq reads are composed of $fastq_{sc}$ and $fastq_{non\text{-}sc}$.

Fig. S10. Effect of lower enzyme concentration on quantitative performance of Quartz-Seq2

We prepared sequence library DNA of Quartz-Seq2 from 10 pg of total RNA of four 384-well plates by using two different reverse-transcription premixes as follows: RT25 (2x Thermopol buffer, 5 units/μL SuperScript III, 0.55 units/μL RNasin plus) and RT6.25 (2x Thermopol buffer, 1.25 units/μL SuperScript III, 0.1375 units/μL RNasin plus) for Quartz-Seq2. We performed four-batch experiments on different experimental days for the RT25 and RT6.25 conditions.

a) We present the quantitative performances of Quartz-Seq2. b) Technical variability of Quartz-Seq2 with RT25 and RT6.25. We present the distribution of gene expression variability from 1,536 wells. We show the mean and S.D of dispersion in a box. c) Quantitative performance of ERCC spike-in RNA for Quartz-Seq2 under two different conditions. The x-axis represents the average of the detection limit. The y-axis represents the ERCC capture efficiency.

Fig. S11. Technical introduction to Drop-seq

a) We observed a clear knee in the cumulative distribution plot of the UMI count, which corresponded to the cell barcodes derived from cells.

b) We validated our technical introduction to Drop-seq using a species mixing experiment. Specifically, we performed a Drop-seq experiment using a mixture of mouse ES cells and human iPS cells. We analyzed 16,000 barcode beads. We confirmed that sequence reads of each cell barcode were exclusively mapped to mouse and human transcripts.

c) Schematic representation of the experimental design for the validation of Drop-seq and Quartz-Seq2. ES cells and Dex-treated ES cells were dissociated from each other. Dex-treated ES cells differentiated into primitive endoderm-like cells. The cell suspensions for these were equally mixed. We used this mixture of cells for Drop-seq and Quartz-Seq2 in the “RT25” condition.

d) Knee plot of UMI count for Drop-seq using a mixture of ES cells and Dex-treated ES cells. We selected 500 STAMPs for downstream analysis of Drop-seq.

e) The number of UMI count and gene count per cell in the selected number of STAMPs.

f) Distribution of gene count for Quartz-Seq2 using ES cells and Dex-treated ES cells. We excluded low-quality cells that expressed fewer than 4,000 genes.

g) Drop-seq and Quartz-Seq2 could distinguish between ES cells and differentiated cells by principal component analysis. It is known that the *Dab2* gene is highly expressed in differentiated cells. Red dots represent differentiated cells that highly express the *Dab2* gene.

h) Schematic representation of filtering before the calculation of differentially expressed genes between ES and Dex-treated ES cells (PrE cells).

Fig. S12. Simulation-based power analysis for Figures 3 and 4

a) We performed simulation-based power analysis by using the data set of Quartz-Seq2 and Drop-seq in Figure 3 with G6GR ES cells. The true positive rate (TPR) and false discovery rate (FDR) were determined for 0.24 million reads on average per cell for the following sample sizes: n = 16, n = 32, n = 64, n = 128, n = 256, and n = 512 per group. Line plots represent the median of 25 simulations. We also show the absolute numbers of true-positive-simulated genes and false-discovery-simulated genes. Line plots represent the median and S.D. of 25 simulations.

b-c) Simulation-based power analysis by using the data set of Quartz-Seq2 and other methods presented in Figure 4 with J1 ES cells. True positive rate (TPR) and false discovery rate (FDR) were determined for 0.1 million reads on average per cell for the following sample sizes: n = 16, n = 32, n = 64, n = 128, n = 256, and n = 512 per group. Line plots represent the median of 25 simulations. We also show the absolute numbers of true-positive-simulated genes and false-discovery-simulated genes. Line plots represent the median and S.D. of 25 simulations.

Fig. S13. Assessment of quantitative performance for external control RNA using Quartz-Seq2 data and previously reported data

a) Quantitative data of ERCC spike-in RNA about Quartz-Seq2 data, including 25 batch experiments corresponding to 11,520-well samples. We plotted the ERCC capture efficiency and average detection limit at respective data points of initial fastq reads for 25 batch experiments. All batch experiments can ensure 30,000 fastq reads/well on average (red line). We calculated the ERCC capture efficiency and detection limit for CEL-seq2(C1), CEL-seq2, and MARS-seq by using a digital expression matrix from Svensson et al. [2]. We observed that these performances strongly correlated with the numbers of initial fastq reads and samples. Nevertheless, Quartz-Seq2 had good performance equivalent to or higher than the range of fewer initial fastq reads.

b) Further comparison of the detection limit between Quartz-Seq2 and CEL-seq(C1). We reanalyzed CEL-seq2(C1) (n=96) with SRA files, which were also used by Svensson et al. [2-3], using our pipeline. First, we confirmed that the detection limit of reanalyzed CEL-seq2(C1) was not an underestimate compared with that of previously analyzed CEL-seq2(C1). We observed

that the detection limit of all Quartz-Seq2 data from 25 batch experiments was lower than that of CEL-seq2 in the range of 30,000 initial fastq reads.

c) Strong correlation between the quantitative performance of ERCC spike-in RNA and the average amount of total RNA per single cell. The x-axis represents the average amount of total RNA per single cell. The y-axis represents the ERCC capture efficiency or average detection limit of ERCC spike-in RNA. We also show Spearman's rank correlation (SCC) between the average amount of total RNA per single cell and the quantitative performances of ERCC spike-in RNA from respective batch experiments.

d) We show the ERCC capture efficiency and detection limit from respective single-cell RNA methods using J1 ES cells.

2. Svensson V, Natarajan KN, Ly L-H, Miragaia RJ, Labalette C, Macaulay IC, et al. Power analysis of single-cell RNA-sequencing experiments. Nat. Methods. 2017;6:150.

3. Hashimshony T, Senderovich N, Avital G, Klochendler A, de Leeuw Y, Anavy L, et al. CEL-Seq2: sensitive highly-multiplexed single-cell RNA-Seq. Genome Biol. 2016;17:892.

Fig. S14. Sequence platform and sequence library preparation for Quartz-Seq2

a) There was no obvious difference between the NextSeq500 platform and the HiSeq2500 platform for Quartz-Seq2. We analyzed the same sequence library DNA with these different platforms. We did not observe any clear differences in UMI count and gene count.

b) We show the relative costs with the three sequence platforms for Quartz-Seq2. Read2 sequence of Quartz-Seq2 was used for the mapping to transcript. A Read2 length of 62 nt in NextSeq500 was the most cost-effective setting for Quartz-Seq2.

c) Read2 length affected the unique mapping ratio. We prepared the three kinds of sequence library DNA from the same amplified cDNA of Quartz-Seq2. We prepared the library DNA using the ligation method and the Nextera method. We also prepared data comprising a mixture of ligation data and Nextera data.

Fig. S15. We did not observe a clear batch effect among the twelve 384-well plates in Quartz-Seq2

We plotted the 4,484 cells on t-SNE space with color labeling for each 384-well PCR plates.

Fig. S16. Quality check of high proportion of mitochondrial RNA for a cell cluster

We show the UMI count, gene count, and the proportion of mitochondrial RNA for each cluster. We present the mean UMI count for each cluster. In cluster 2, both marker genes (ES and PrE marker genes) were expressed. In addition, the UMI count of cluster 2 was about twice those of clusters 1 and 4. Therefore, cluster 2 was judged to represent doublets of an ES cell and a PrE cell. Moreover, we determined the UMI count derived from mitochondrial genes. We also calculated the proportion of mitochondrial RNA for respective cell clusters. In cluster 6, the proportion was higher than for the other cell clusters.

Fig. S17. Magnified version of Figure 6c

Fig. S18. Quartz-Seq2 with ultra-shallow fastq reads enables identification of heterogeneity of cell cycle phase within a cell type.

a) Cells are plotted on t-SNE space with coloring representing the intensity of Hoechst 33342 staining. We used 0.01 M fastq reads on average per cell as initial reads. b) Identification of cell cycle-associated genes.

Fig. S19. Correlation between gene count and cell size in SVF populations

a) Distribution of gene counts from respective single cells. We excluded those single cells expressing 500 or less different genes. b) Correlation between gene count and cell size from flow cytometry data. Each dot represents a single cell. Different colors are used to identify the different cell clusters. c) Distribution of cell size for each cell cluster. d) Immunofluorescence images of stromal vascular fraction. Yellow allow-heads represent stained cell with antibody. White scale bars represent 10 μm.

Fig. S20. Full-sized image of Figure 7d

Fig. S21. Expression profile of ligand and receptor genes for stromal vascular fraction

We present the gene expression using Quartz-Seq2 data and previous reported list of ligand and receptor genes [4]. Listed gene expression is displayed as a blue color in a heatmap. Class labeling of single-cells in stromal vascular fraction is displayed as each color. a) Heatmap of gene expression for ligand gene. b) Heatmap of gene expression for receptor gene.

4. Ramilowski JA, Goldberg T, Harshbarger J, Kloppman E, Lizio M, Satagopam VP, et al. A draft network of ligand–receptor-mediated multicellular signalling in human. Nat Commun. 2015;6:7866.

Fig. S22. Expression profile of previously reported MSC marker genes for stromal vascular fraction

We present the gene expression using Quartz-Seq2 data. We used a previously reported list of marker genes (*Thy1/Cd90, Eng/Cd105, Pdgfra, Lys6a/Sca1, Tnnt2, Nanog, Pou5f1, Sox2, Myog, Cd34, Cd31, Cd45*) in MSCs.

Fig. S23. Expression profile of previously reported ASC marker genes for clusters 1 and 8

We present the gene expression using Quartz-Seq2 data. We used a previously reported list of marker genes in CD34+/CD55+/Dpp4+ adipose-derived stem cells (ASC). Almost all marker genes were enriched in cluster 1.

Fig. S24. Definition of “variable genes the expression of which is less associated with the cell cycle phase”

a) In our strategy, cells were discretized into 40 equal-sized buckets based on the rank of Hoechst 33342 staining intensity. Then, the CV of averaged UMI counts for a gene in each bin was calculated (as mentioned in Methods). b) We represented the definition of “variable genes the expression of which is less associated with the cell cycle phase”.

Fig. S1

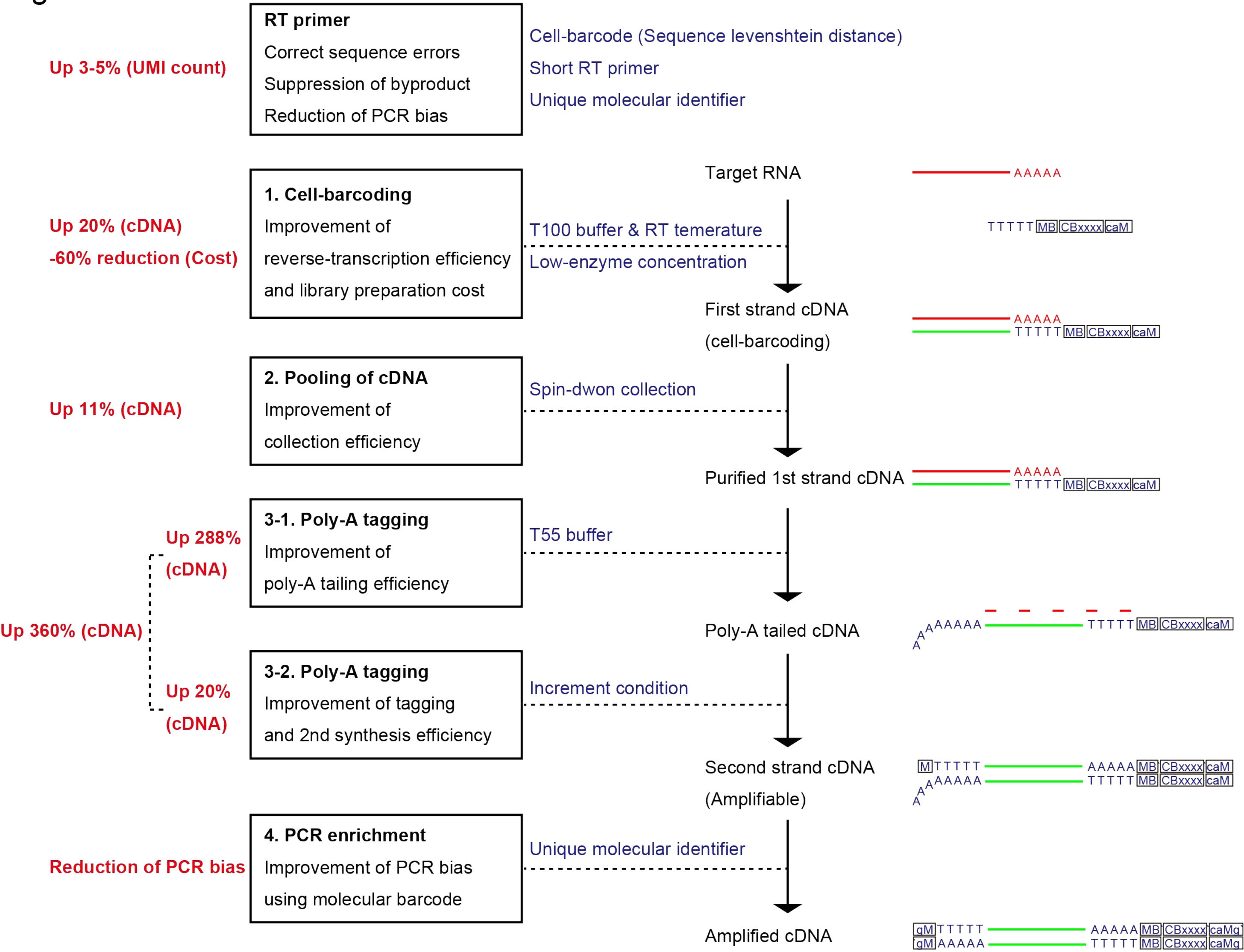

Up 3-5% (UMI count)
RT primer
Correct sequence errors
Suppression of byproduct
Reduction of PCR bias
Cell-barcode (Sequence levenshtein distance)
Short RT primer
Unique molecular identifier
Target RNA
AAAAA
Up 20% (cDNA)
-60% reduction (Cost)
1. Cell-barcoding
Improvement of
reverse-transcription efficiency
and library preparation cost
T100 buffer & RT temerature
Low-enzyme concentration
TTTTT MB CBxxxx caM
First strand cDNA
(cell-barcoding)
Up 11% (cDNA)
2. Pooling of cDNA
Improvement of
collection efficiency
Spin-dwon collection
Purified 1st strand cDNA
Up 288%
(cDNA)
3-1. Poly-A tagging
Improvement of
poly-A tailing efficiency
T55 buffer
Up 360% (cDNA)
Poly-A tailed cDNA
Up 20%
(cDNA)
3-2. Poly-A tagging
Improvement of tagging
and 2nd synthesis efficiency
Increment condition
Second strand cDNA
(Amplifiable)
Reduction of PCR bias
4. PCR enrichment
Improvement of PCR bias
using molecular barcode
Unique molecular identifier
Amplified cDNA
gM TTTTT
AAAAA MB CBxxxx caMg

Fig. S2

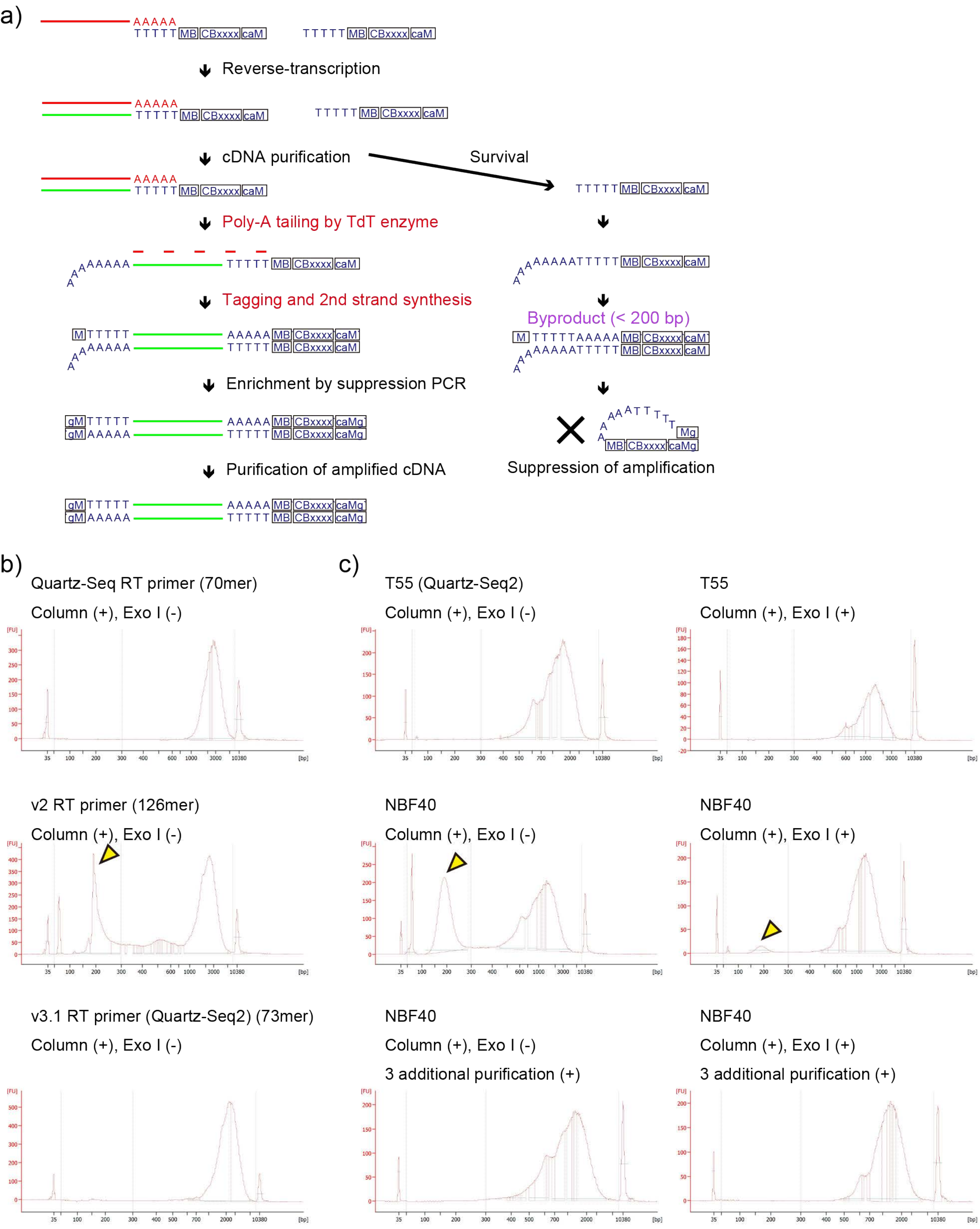
a)
AAAAA
TTTTT MB CBxxxx caM
TTTTT MB CBxxxx caM
Reverse-transcription
cDNA purification
Survival
Poly-A tailing by TdT enzyme
Tagging and 2nd strand synthesis
Byproduct (< 200 bp)
Enrichment by suppression PCR
Suppression of amplification
Purification of amplified cDNA
b)
Quartz-Seq RT primer (70mer)
Column (+), Exo I (-)
v2 RT primer (126mer)
Column (+), Exo I (-)
v3.1 RT primer (Quartz-Seq2) (73mer)
Column (+), Exo I (-)
c)
T55 (Quartz-Seq2)
Column (+), Exo I (-)
T55
Column (+), Exo I (+)
NBF40
Column (+), Exo I (-)
NBF40
Column (+), Exo I (+)
NBF40
Column (+), Exo I (-)
3 additional purification (+)
NBF40
Column (+), Exo I (+)
3 additional purification (+)
[FU]
[bp]

Fig. S3

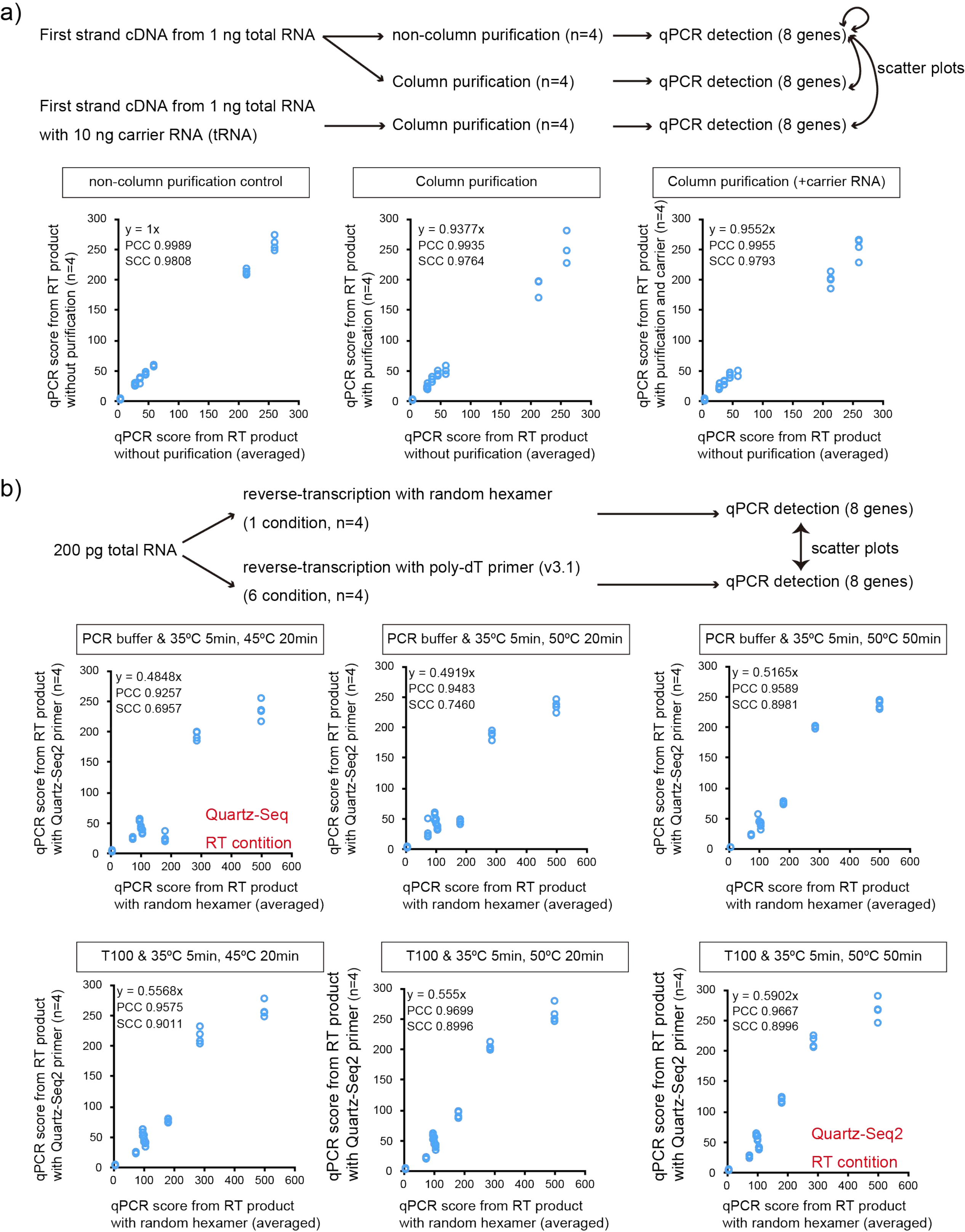
a)
First strand cDNA from 1 ng total RNA
non-column purification (n=4)
Column purification (n=4)
First strand cDNA from 1 ng total RNA
with 10 ng carrier RNA (tRNA)
Column purification (n=4)
qPCR detection (8 genes)
qPCR detection (8 genes)
qPCR detection (8 genes)
scatter plots
non-column purification control
y = 1x
PCC 0.9989
SCC 0.9808
qPCR score from RT product without purification (n=4)
qPCR score from RT product without purification (averaged)
Column purification
y = 0.9377x
PCC 0.9935
SCC 0.9764
qPCR score from RT product with purification (n=4)
qPCR score from RT product without purification (averaged)
Column purification (+carrier RNA)
y = 0.9552x
PCC 0.9955
SCC 0.9793
qPCR score from RT product with purification and carrier (n=4)
qPCR score from RT product without purification (averaged)
b)
200 pg total RNA
reverse-transcription with random hexamer
(1 condition, n=4)
reverse-transcription with poly-dT primer (v3.1)
(6 condition, n=4)
qPCR detection (8 genes)
qPCR detection (8 genes)
scatter plots
PCR buffer & 35ºC 5min, 45ºC 20min
y = 0.4848x
PCC 0.9257
SCC 0.6957
Quartz-Seq
RT contition
PCR buffer & 35ºC 5min, 50ºC 20min
y = 0.4919x
PCC 0.9483
SCC 0.7460
PCR buffer & 35ºC 5min, 50ºC 50min
y = 0.5165x
PCC 0.9589
SCC 0.8981
T100 & 35ºC 5min, 45ºC 20min
y = 0.5568x
PCC 0.9575
SCC 0.9011
T100 & 35ºC 5min, 50ºC 20min
y = 0.555x
PCC 0.9699
SCC 0.8996
T100 & 35ºC 5min, 50ºC 50min
y = 0.5902x
PCC 0.9667
SCC 0.8996
Quartz-Seq2
RT contition
qPCR score from RT product with Quartz-Seq2 primer (n=4)
qPCR score from RT product with random hexamer (averaged)

Fig. S4

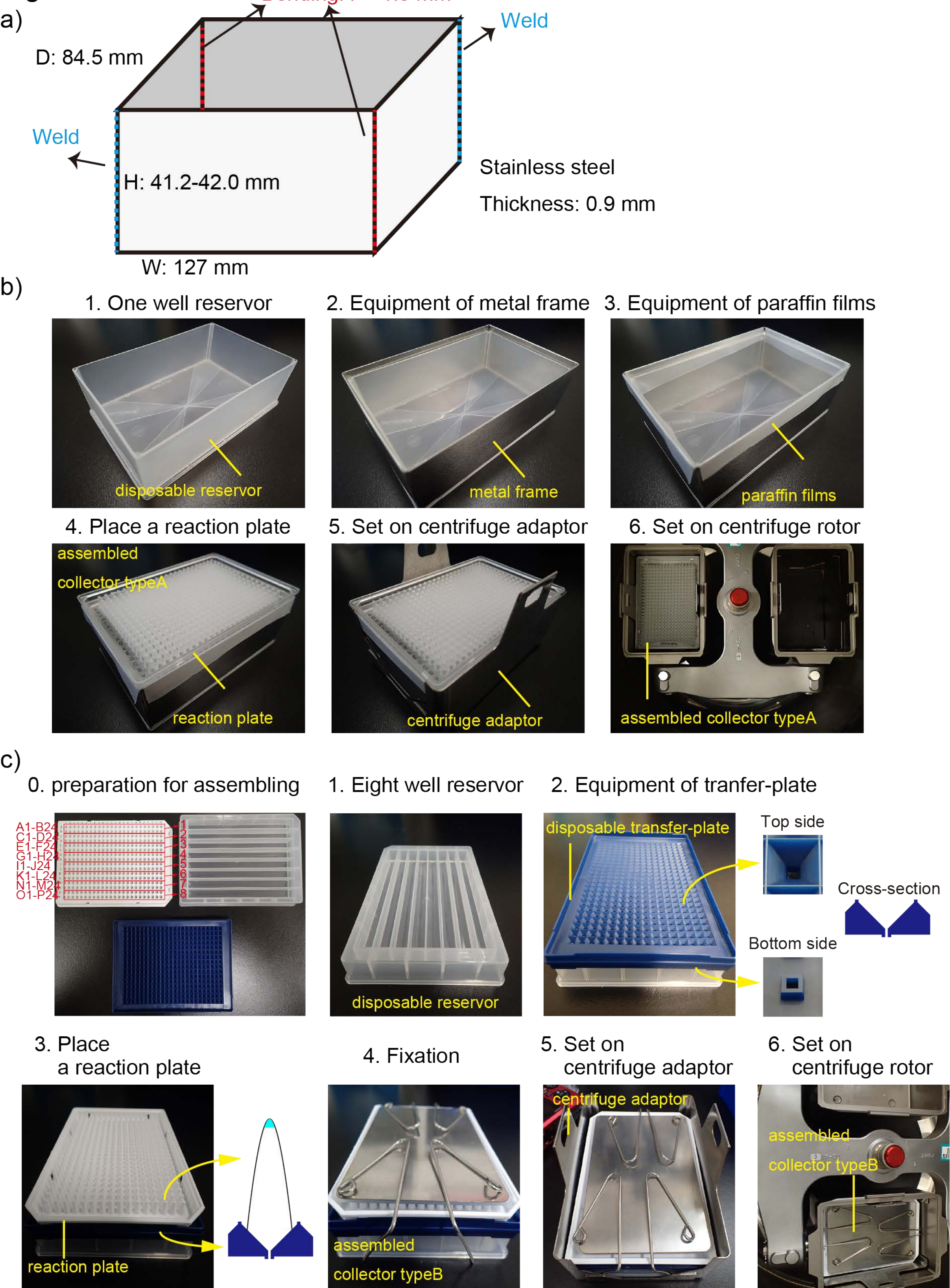

a)
Bending: r = 1.5 mm
Weld
D: 84.5 mm
Weld
H: 41.2-42.0 mm
Stainless steel
Thickness: 0.9 mm
W: 127 mm
b)
1. One well reservor
disposable reservor
2. Equipment of metal frame
metal frame
3. Equipment of paraffin films
paraffin films
4. Place a reaction plate
assembled
collector typeA
reaction plate
5. Set on centrifuge adaptor
centrifuge adaptor
6. Set on centrifuge rotor
assembled collector typeA
c)
0. preparation for assembling
A1-B24
C1-D24
E1-F24
G1-H24
I1-J24
K1-L24
N1-M24
O1-P24
1
2
3
4
5
6
7
8
1. Eight well reservor
disposable reservor
2. Equipment of tranfer-plate
disposable transfer-plate
Top side
Cross-section
Bottom side
3. Place
a reaction plate
reaction plate
4. Fixation
assembled
collector typeB
5. Set on
centrifuge adaptor
centrifuge adaptor
6. Set on
centrifuge rotor
assembled
collector typeB

Fig. S5

a)

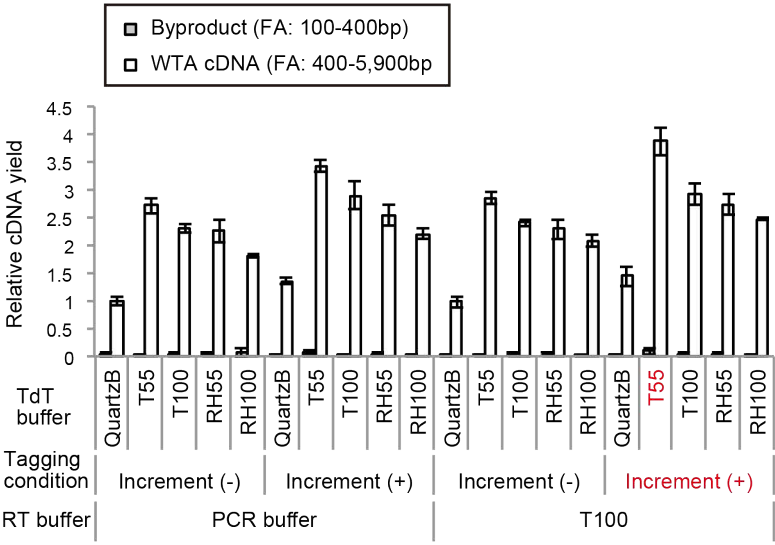

Byproduct (FA: 100-400bp)
WTA cDNA (FA: 400-5,900bp
Relative cDNA yield
4.5
4
3.5
3
2.5
2
1.5
1
0.5
0
TdT buffer
QuartzB
T55
T100
RH55
RH100
QuartzB
T55
T100
RH55
RH100
QuartzB
T55
T100
RH55
RH100
QuartzB
T55
T100
RH55
RH100
Tagging condition
Increment (-)
Increment (+)
Increment (-)
Increment (+)
RT buffer
PCR buffer
T100

b)

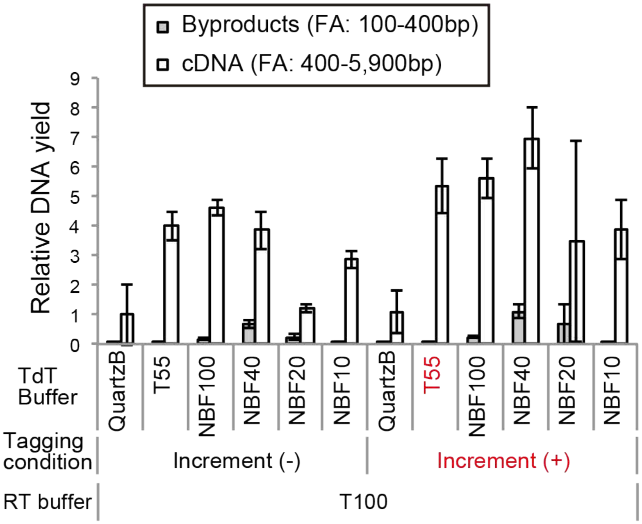

Byproducts (FA: 100-400bp)
cDNA (FA: 400-5,900bp)
Relative DNA yield
9
8
7
6
5
4
3
2
1
0
TdT Buffer
QuartzB
T55
NBF100
NBF40
NBF20
NBF10
QuartzB
T55
NBF100
NBF40
NBF20
NBF10
Tagging condition
Increment (-)
Increment (+)
RT buffer
T100

c)

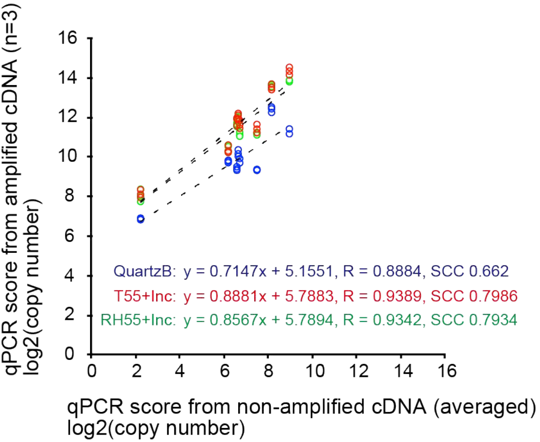

qPCR score from amplified cDNA (n=3) log2(copy number)
16
14
12
10
8
6
4
2
0
QuartzB: y = 0.7147x + 5.1551, R = 0.8884, SCC 0.662
T55+Inc: y = 0.8881x + 5.7883, R = 0.9389, SCC 0.7986
RH55+Inc: y = 0.8567x + 5.7894, R = 0.9342, SCC 0.7934
0
2
4
6
8
10
12
14
16
qPCR score from non-amplified cDNA (averaged) log2(copy number)

Fig. S6

a)

Data size	Platform	For Quartz-Seq2 Indexes	For Quartz-Seq2 Condition	Method
0.1M fastq reads per single-cell	Cell sorter			Quartz-Seq
	Fluidigm C1			CEL-seq2 (C1)
	Cell sorter			CEL-seq2
	Cell sorter			SCRB-seq
	Cell sorter			MARS-seq
	Cell sorter	384	RT100	Quartz-Seq2
	Cell sorter	384	RT25	Quartz-Seq2
	Cell sorter	384	RT6.25	Quartz-Seq2
	Cell sorter	384	RT6.25+C1	Quartz-Seq2
	Cell sorter	1,536	RT25	Quartz-Seq2
	Cell sorter	1,536	RT6.25+C3	Quartz-Seq2
	Droplet			Drop-seq
0.2M fastq reads per single-cell	Cell sorter			Quartz-Seq
	Fluidigm C1			CEL-seq2 (C1)
	Cell sorter			CEL-seq2
	Cell sorter			SCRB-seq
	Cell sorter			MARS-seq
	Cell sorter	384	RT100	Quartz-Seq2
	Cell sorter	384	RT25	Quartz-Seq2
	Cell sorter	384	RT6.25	Quartz-Seq2
	Cell sorter	384	RT6.25+C1	Quartz-Seq2
	Cell sorter	1,536	RT25	Quartz-Seq2
	Cell sorter	1,536	RT6.25+C3	Quartz-Seq2
	Droplet			Drop-seq

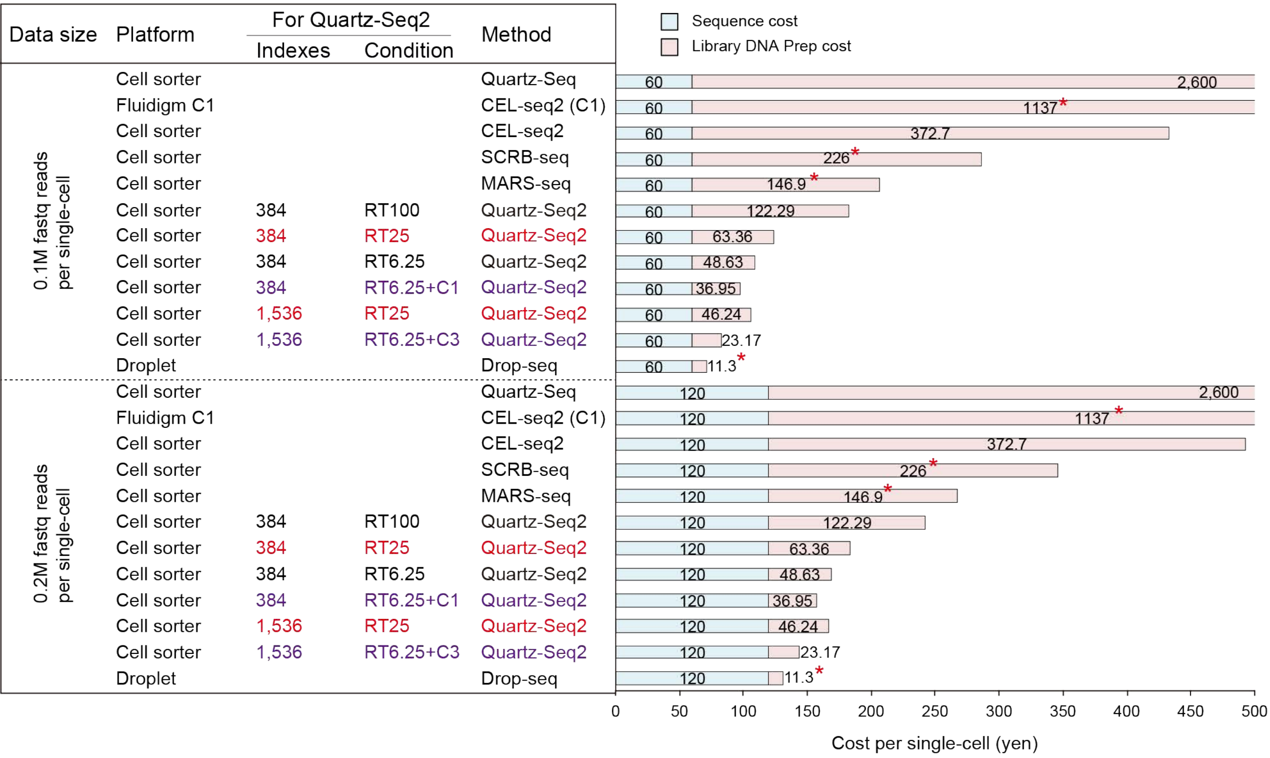

b)

Data size	Platform	For Quartz-Seq2 Indexes	For Quartz-Seq2 Condition	Method
0.1M fastq reads per single-cell	Cell sorter			Quartz-Seq
	Fluidigm C1			CEL-seq2 (C1)
	Cell sorter			CEL-seq2
	Cell sorter			SCRB-seq
	Cell sorter			MARS-seq
	Cell sorter	384	RT100	Quartz-Seq2
	Cell sorter	384	RT25	Quartz-Seq2
	Cell sorter	384	RT6.25	Quartz-Seq2
	Cell sorter	384	RT6.25+C1	Quartz-Seq2
	Cell sorter	1,536	RT25	Quartz-Seq2
	Cell sorter	1,536	RT6.25+C3	Quartz-Seq2
	Droplet			Drop-seq
0.2M fastq reads per single-cell	Cell sorter			Quartz-Seq
	Fluidigm C1			CEL-seq2 (C1)
	Cell sorter			CEL-seq2
	Cell sorter			SCRB-seq
	Cell sorter			MARS-seq
	Cell sorter	384	RT100	Quartz-Seq2
	Cell sorter	384	RT25	Quartz-Seq2
	Cell sorter	384	RT6.25	Quartz-Seq2
	Cell sorter	384	RT6.25+C1	Quartz-Seq2
	Cell sorter	1,536	RT25	Quartz-Seq2
	Cell sorter	1,536	RT6.25+C3	Quartz-Seq2
	Droplet			Drop-seq

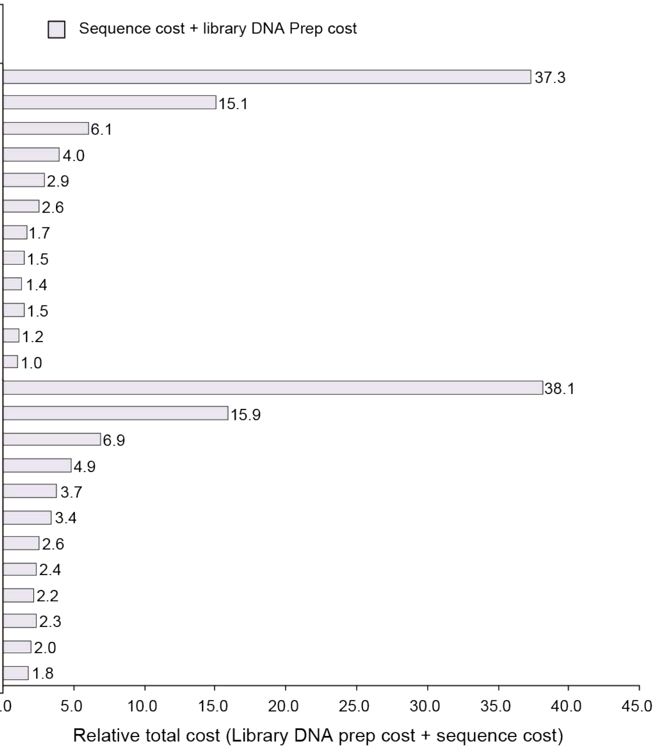

Fig. S7

a)

Method	indexes	RT enzyme concentration (name, U/cell)	The number of column for cDNA purification
Quartz-Seq2	384	RT100, 20U/cell	3
* Quartz-Seq2	384	RT25, 5U/cell	3
Quartz-Seq2	384	RT6.25, 1.25U/cell	3
Quartz-Seq2	384	RT6.25, 1.25U/cell	1
* Quartz-Seq2	1,536	RT25, 5U/cell	8
Quartz-Seq2	1,536	RT6.25, 1.25U/cell	3

* mainly used in this study

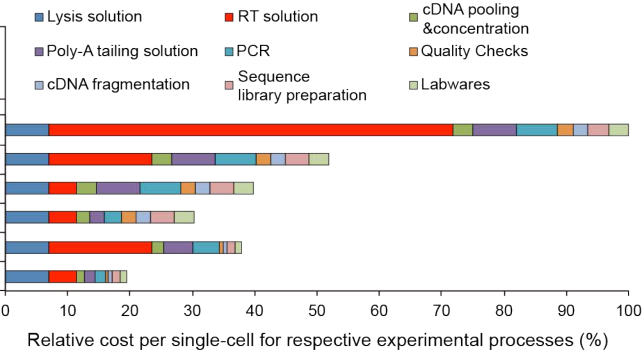

b)

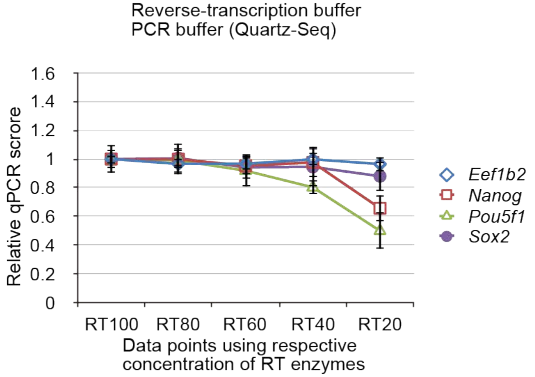

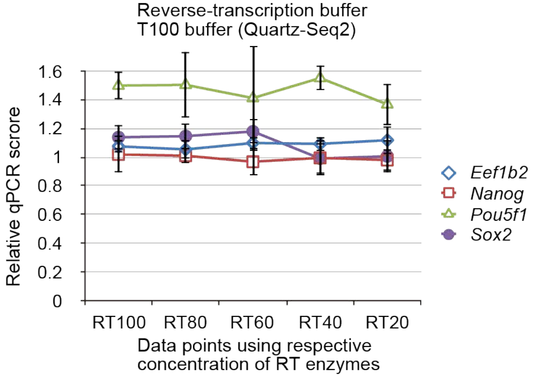

c)

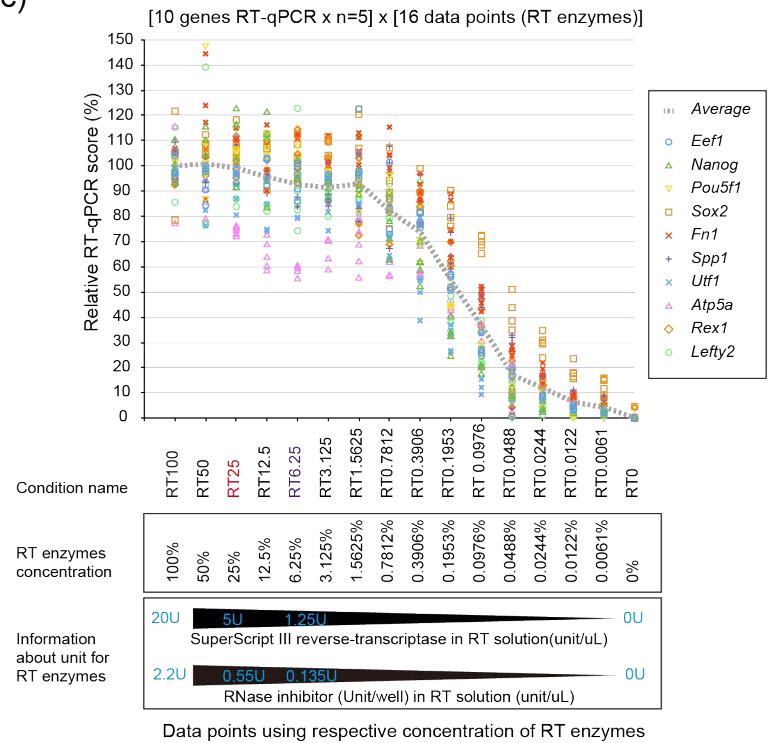

d)

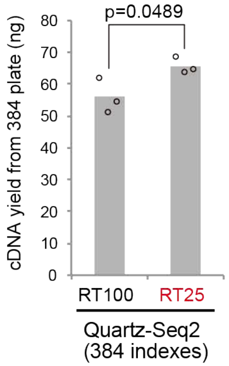

Fig. S8

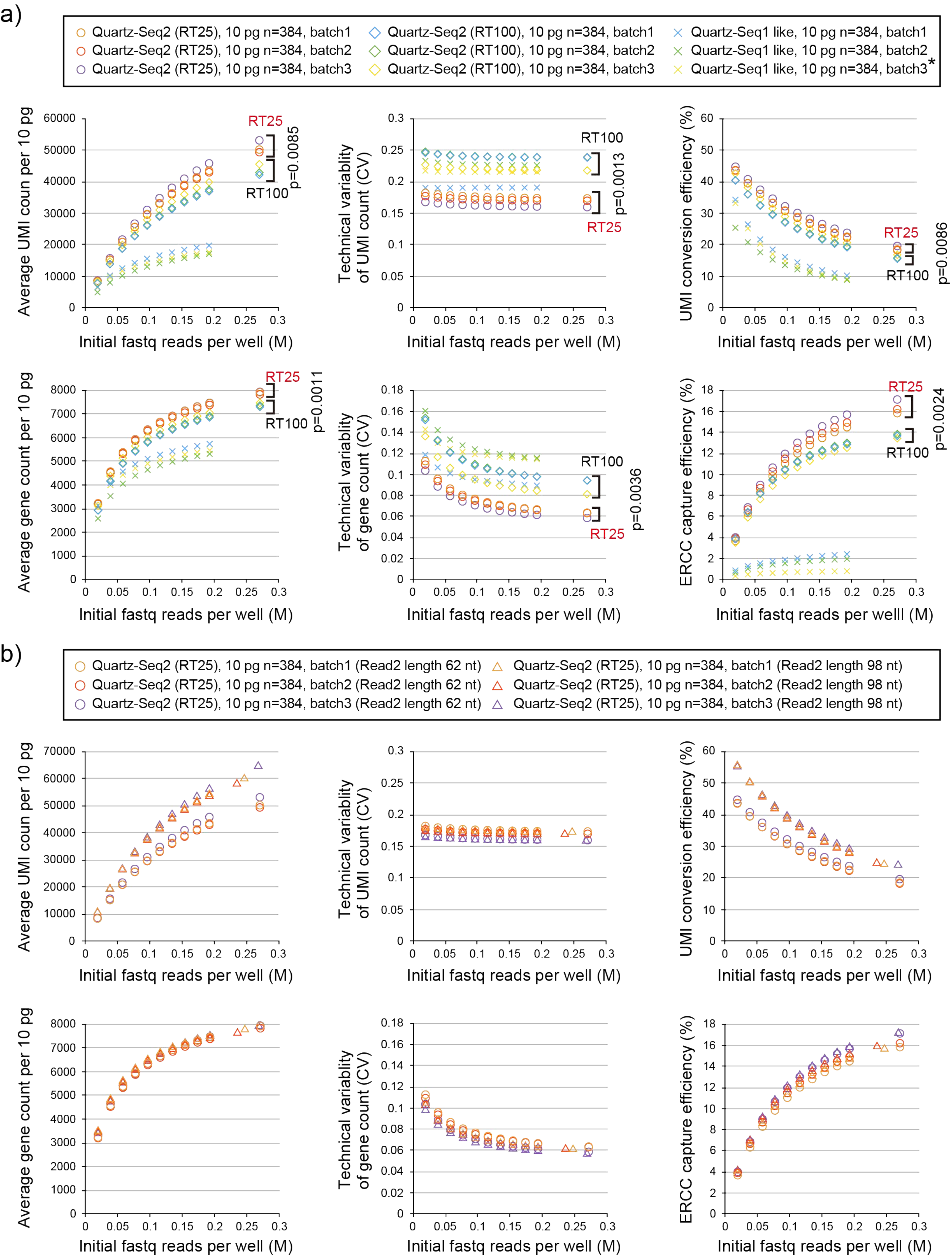
a)
Quartz-Seq2 (RT25), 10 pg n=384, batch1
Quartz-Seq2 (RT25), 10 pg n=384, batch2
Quartz-Seq2 (RT25), 10 pg n=384, batch3
Quartz-Seq2 (RT100), 10 pg n=384, batch1
Quartz-Seq2 (RT100), 10 pg n=384, batch2
Quartz-Seq2 (RT100), 10 pg n=384, batch3
Quartz-Seq1 like, 10 pg n=384, batch1
Quartz-Seq1 like, 10 pg n=384, batch2
Quartz-Seq1 like, 10 pg n=384, batch3*
Average UMI coun per 10 pg
RT25
RT100
p=0.0085
Technical variability of UMI count (CV)
RT100
RT25
p=0.0013
UMI conversion efficiency (%)
RT25
RT100
p=0.0086
Average gene count per 10 pg
RT25
RT100
p=0.0011
Technical variablity of gene count (CV)
RT100
RT25
p=0.0036
ERCC capture efficiency (%)
RT25
RT100
p=0.0024
Initial fastq reads per well (M)
Initial fastq reads per welll (M)
b)
Quartz-Seq2 (RT25), 10 pg n=384, batch1 (Read2 length 62 nt)
Quartz-Seq2 (RT25), 10 pg n=384, batch2 (Read2 length 62 nt)
Quartz-Seq2 (RT25), 10 pg n=384, batch3 (Read2 length 62 nt)
Quartz-Seq2 (RT25), 10 pg n=384, batch1 (Read2 length 98 nt)
Quartz-Seq2 (RT25), 10 pg n=384, batch2 (Read2 length 98 nt)
Quartz-Seq2 (RT25), 10 pg n=384, batch3 (Read2 length 98 nt)
Average UMI coun per 10 pg
Technical variability of UMI count (CV)
UMI conversion efficiency (%)
Average gene count per 10 pg
Technical variablity of gene count (CV)
ERCC capture efficiency (%)
Initial fastq reads per well (M)

Fig. S9

$$\text{UMI conversion efficiency} = \frac{\sum UMI_{sc}}{\sum fastq_{sc} + fastq_{non\text{-}sc}}$$

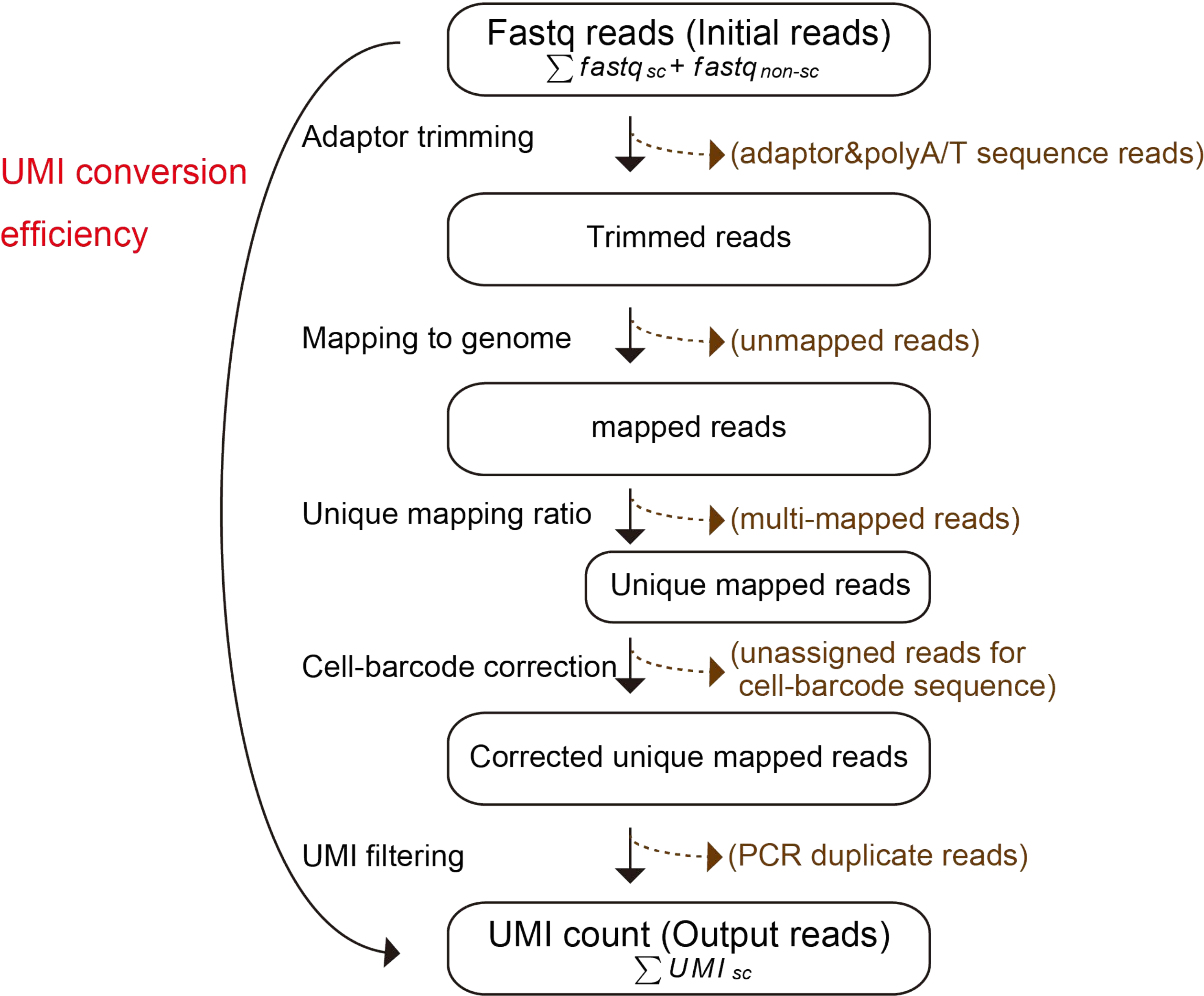

Fig. S10

a)

Sample name	Sample	Method	Type of indexes	Concentration of RT enzymes (%)	Experimental Day & batch number	The number of initial fastq reads per well (on average)
RT25_mES10pgV31_p24	mouse 10 pg total RNA (n=384)	Quartz-Seq2	384	25	Day 1 & batch 1	100,000
RT25_mES10pgV31_p25	mouse 10 pg total RNA (n=384)	Quartz-Seq2	384	25	Day 1 & batch 2	100,000
RT25_mES10pgV31_p28	mouse 10 pg total RNA (n=384)	Quartz-Seq2	384	25	Day 2 & batch 3	100,000
RT25_mES10pgV31_p29	mouse 10 pg total RNA (n=384)	Quartz-Seq2	384	25	Day 2 & batch 4	100,000
RT625_mES10pgV31_p22	mouse 10 pg total RNA (n=384)	Quartz-Seq2	384	6.25	Day 1 & batch 1	100,000
RT625_mES10pgV31_p23	mouse 10 pg total RNA (n=384)	Quartz-Seq2	384	6.25	Day 1 & batch 2	100,000
RT625_mES10pgV31_p26	mouse 10 pg total RNA (n=384)	Quartz-Seq2	384	6.25	Day 2 & batch 3	100,000
RT625_mES10pgV31_p27	mouse 10 pg total RNA (n=384)	Quartz-Seq2	384	6.25	Day 2 & batch 4	100,000

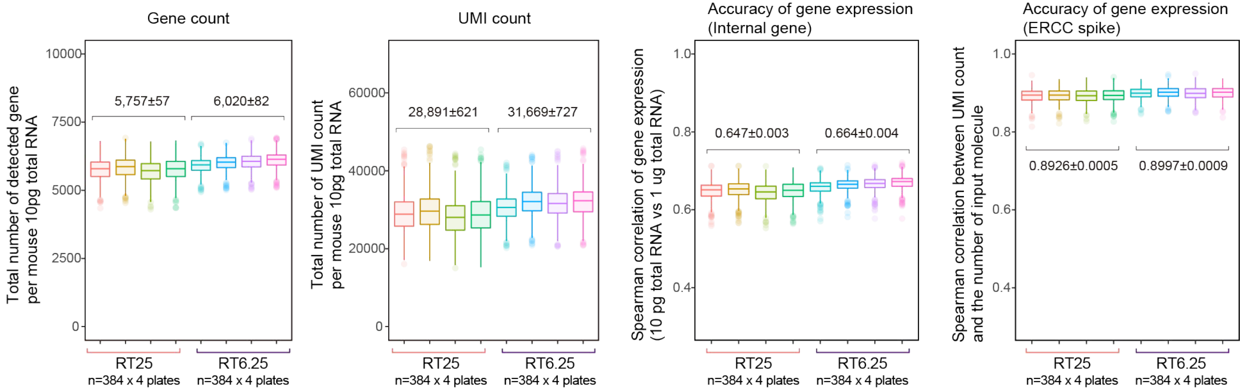

b)

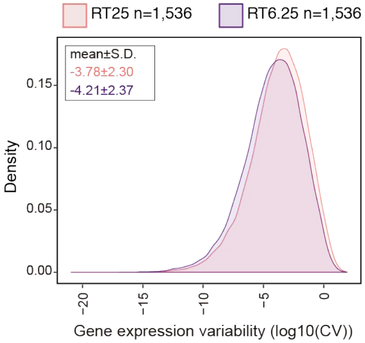

c)

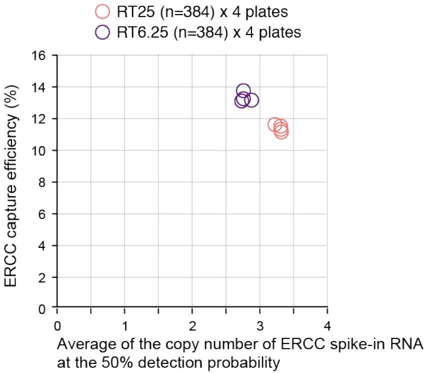

Fig. S11

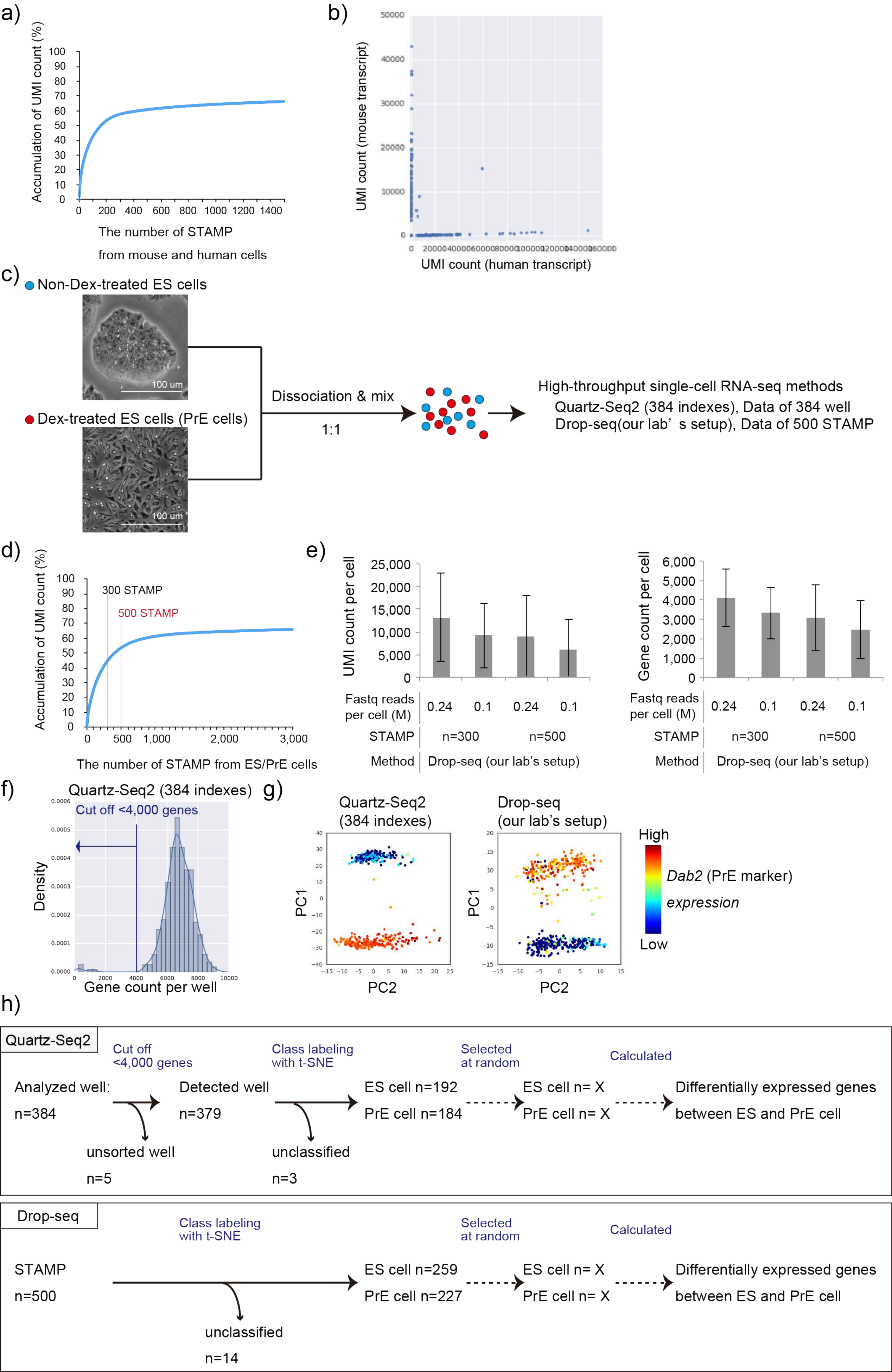
a)
Accumulation of UMI count (%)
The number of STAMP
from mouse and human cells
b)
UMI count (mouse transcript)
UMI count (human transcript)
c)
Non-Dex-treated ES cells
100 um
Dex-treated ES cells (PrE cells)
100 um
Dissociation & mix
1:1
High-throughput single-cell RNA-seq methods
Quartz-Seq2 (384 indexes), Data of 384 well
Drop-seq(our lab' s setup), Data of 500 STAMP
d)
Accumulation of UMI count (%)
300 STAMP
500 STAMP
The number of STAMP from ES/PrE cells
e)
UMI count per cell
Gene count per cell
Fastq reads per cell (M)
0.24
0.1
0.24
0.1
STAMP
n=300
n=500
Method
Drop-seq (our lab's setup)
f)
Quartz-Seq2 (384 indexes)
Cut off <4,000 genes
Density
Gene count per well
g)
Quartz-Seq2 (384 indexes)
Drop-seq (our lab's setup)
PC1
PC2
High
Dab2 (PrE marker) expression
Low
h)
Quartz-Seq2
Cut off <4,000 genes
Class labeling with t-SNE
Selected at random
Calculated
Analyzed well: n=384
Detected well n=379
ES cell n=192
PrE cell n=184
ES cell n= X
PrE cell n= X
Differentially expressed genes between ES and PrE cell
unsorted well n=5
unclassified n=3
Drop-seq
Class labeling with t-SNE
Selected at random
Calculated
STAMP n=500
ES cell n=259
PrE cell n=227
ES cell n= X
PrE cell n= X
Differentially expressed genes between ES and PrE cell
unclassified n=14

Fig. S12

a)

Method	Sample	Data	Related figure
Drop-seq	G6GR ES cell (non-dex treated) n=192	Our data	Figure 3
Quartz-Seq2	G6GR ES cell (non-dex treated) n=259	Our data	Figure 3

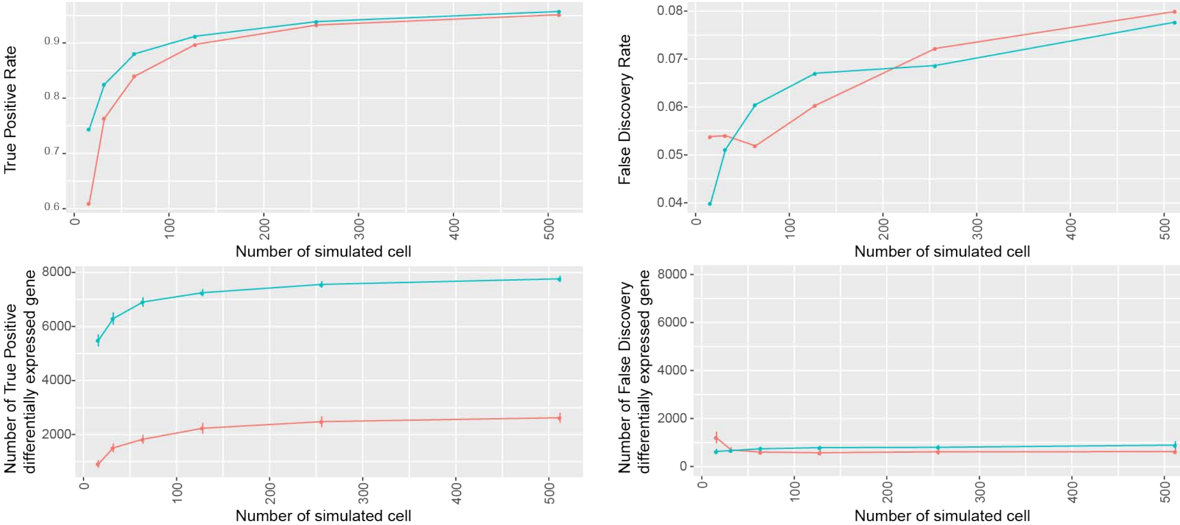

b)

Method	Sample	Data	Related figure
Quartz-Seq2 (RT25) Day1	J1 ES cell (2i/LIF) n=1,152	Our data	Figure 4
Quartz-Seq2 (RT25) Day2	J1 ES cell (2i/LIF) n=768	Our data	Figure 4
Quartz-Seq2 (RT6.25, Column1)	J1 ES cell (2i/LIF) n=768	Our data	Figure 4
CEL-seq2	J1 ES cell (2i/LIF) n=71	*Ziegenhain et al.*2017	Figure 4
SCRB-seq	J1 ES cell (2i/LIF) n=84	*Ziegenhain et al.*2017	Figure 4
MARS-seq	J1 ES cell (2i/LIF) n=65	*Ziegenhain et al.*2017	Figure 4
Drop-seq	J1 ES cell (2i/LIF) n=76	*Ziegenhain et al.*2017	Figure 4

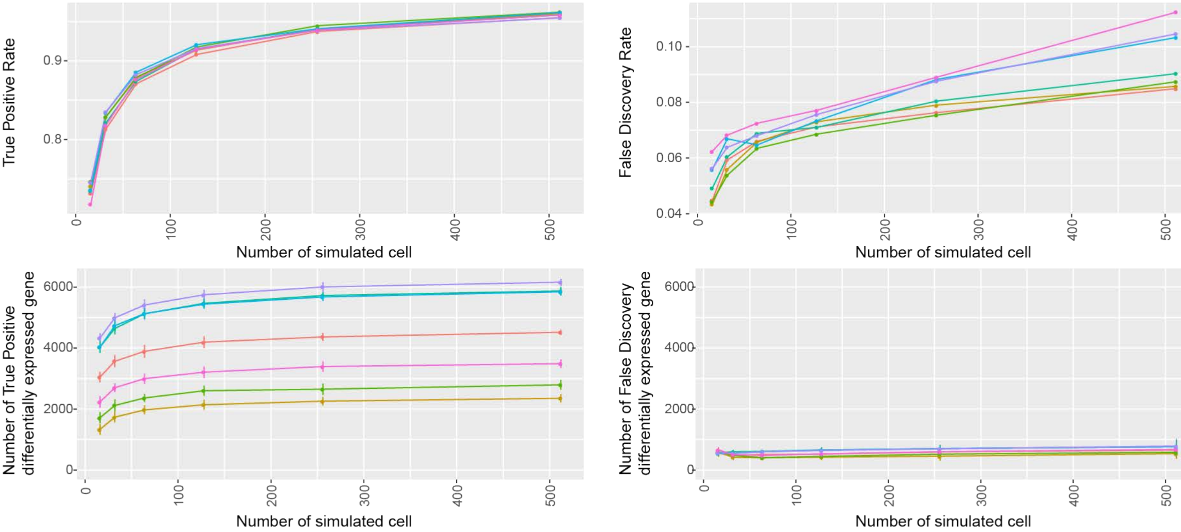

c)

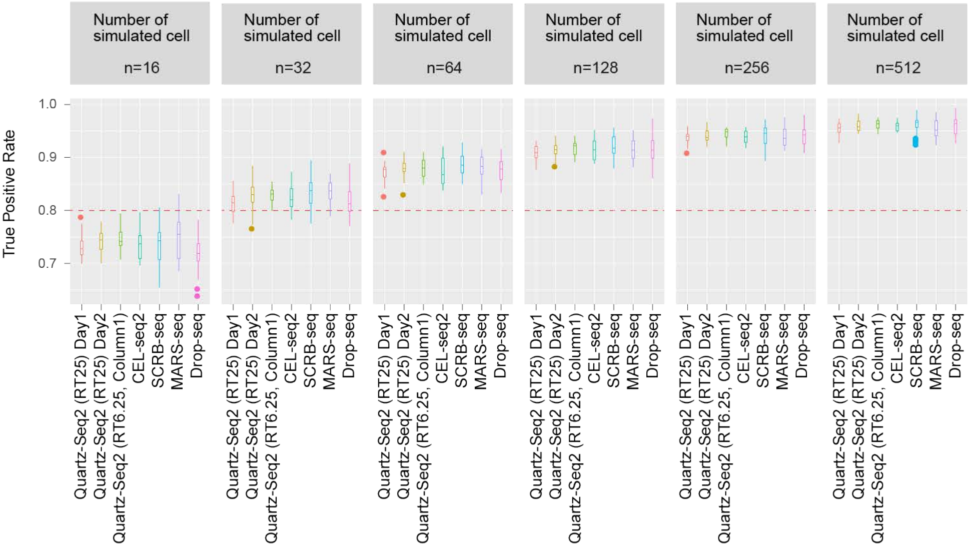

Fig. S13

a)

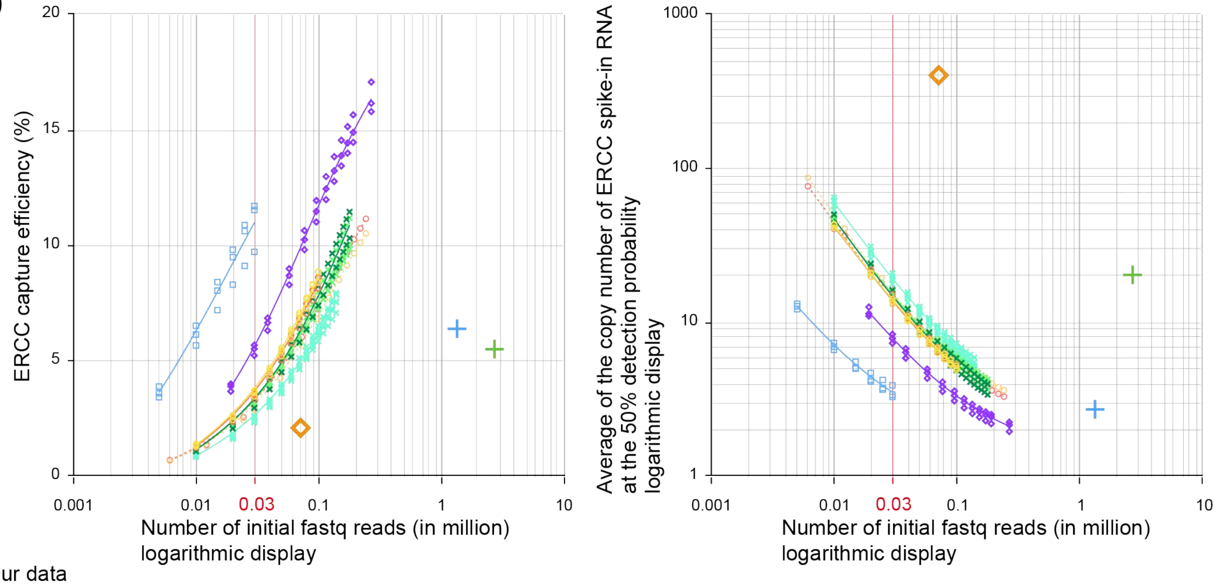

Our data

Platfom	Cell-type	Method	RT condition	Type of Index	Cost of library DNA preparation
Cell-sorter	10 pg total RNA	Quartz-Seq2 n=1,152<br>3 batches (384 well x 3 plates)	RT25	384 indexes	¥61.3-63.3/well
Cell-sorter	Stromal vascular fraction	Quartz-Seq2 n=1,152<br>3 batches (384 well x 3 plates)	RT25	384 indexes	¥61.3-63.3/well
Cell-sorter	G6GR ES cell	Quartz-Seq2 n=192<br>1 batch (384 well x 0.5 x 1 plate)	RT25	384 indexes	¥61.3-63.3/well
Cell-sorter	Dex-treated<br>G6GR ES cell (PrE)	Quartz-Seq2 n=192<br>1 batch (384 well x 0.5 x 1 plate)	RT25	384 indexes	¥61.3-63.3/well
Cell-sorter	G6GR ES cell	Quartz-Seq2 n=2,304<br>3 batches (384 well x 0.5 x 12 plates)	RT25	1,536 indexes	¥45.7-46.2/well
Cell-sorter	Dex-treated<br>G6GR ES cell (PrE)	Quartz-Seq2 n=2,304<br>3 batches (384 well x 0.5 x 12 plates)	RT25	1,536 indexes	¥45.7-46.2/well
Cell-sorter	J1 ES cell (2i/LIF)	Quartz-Seq2 n=2,304<br>6 batches (384 well x 6 plates)	RT25	384 indexes	¥61.3-63.3/well
Cell-sorter	J1 ES cell (2i/LIF)	Quartz-Seq2 n=1,152<br>3 batches (384 well x 3 plates)	RT6.25	384 indexes	¥46.6-48.6/well
Cell-sorter	J1 ES cell (2i/LIF)	Quartz-Seq2 n=768<br>2 batches (384 well x 2 plates)	RT6.25<br>&Column1	384 indexes	¥34.9-36.9/well

Data (digital expression matrix) from *Svensson et al. Nat. Methods. 2017;6:150.*

Platfom	Cell-type	Method	Original data (Accession ID & Reference)
Fluidigm C1	Fibroblast	CEL-seq2(C1) n=96	GSE78779 SRP070989, *Hashimshony et al 2016*
Cell-sorter	Fibroblast	CEL-seq2 n=44	GSE78779 SRP070989, *Hashimshony et al 2016*
Cell-sorter	Dendritic cell	MARS-seq n=384	GSE54006 SRP035326, *Jaitin et al 2014*

b)

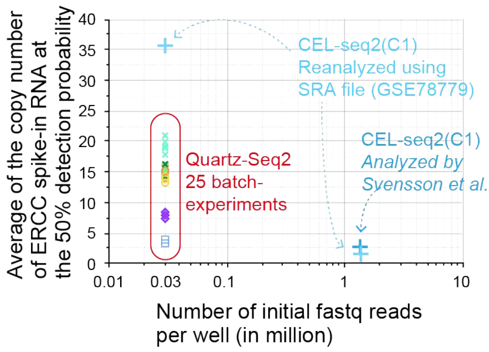

c) [Quartz-Seq2 (RT25), 0.03 million fastq reads/cell (on average)]

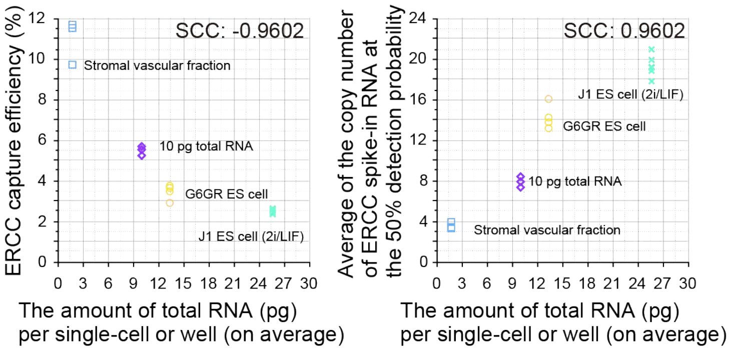

d)

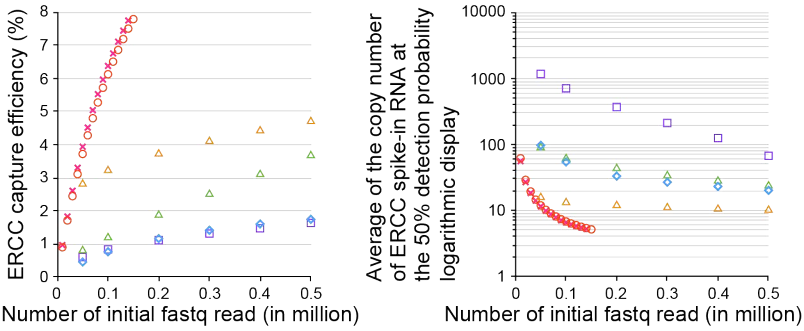

J1 ES cell 2i/LIF, HiSeq platform	
Our data	Quartz-Seq2 day1 (n=1,152)<br>Quartz-Seq2 day2 (n=768)
Data from *Ziegenhain et al.2017*	CEL-seq2(C1) (n=71)<br>SCRB-seq (n=84)<br>MARS-seq (n=65)<br>Drop-seq (w/o ERCC) (n=76)

without cell (ERCC data), NextSeq platform	
Data from *Macosko et al. 2015*	Drop-seq (w ERCC) (n=75)

Fig. S14

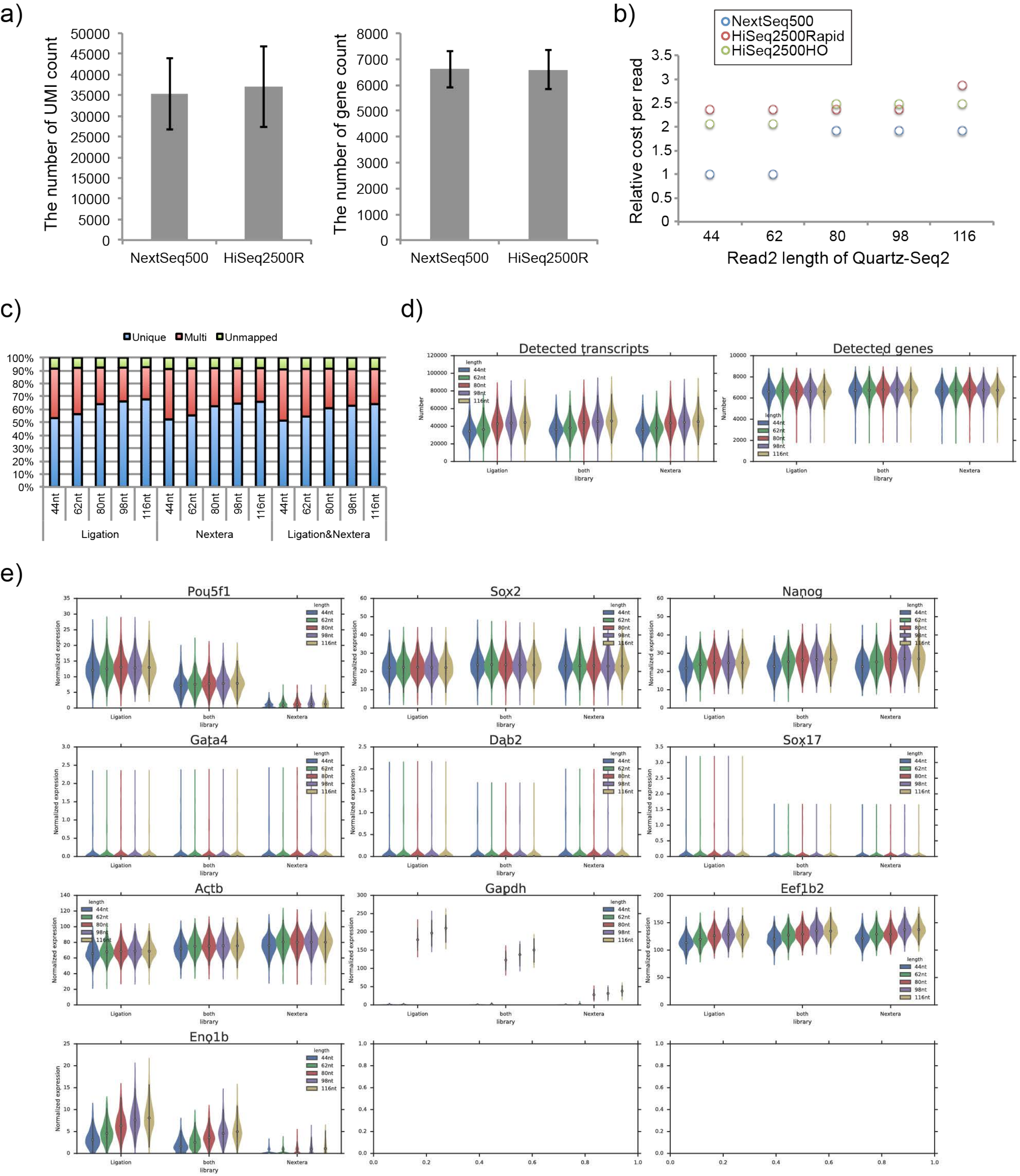

a)
The number of UMI count
NextSeq500
HiSeq2500R
The number of gene count
b)
NextSeq500
HiSeq2500Rapid
HiSeq2500HO
Relative cost per read
Read2 length of Quartz-Seq2
c)
Unique
Multi
Unmapped
Ligation
Nextera
Ligation&Nextera
d)
Detected transcripts
Detected genes
e)
Pou5f1
Sox2
Nanog
Gata4
Dab2
Sox17
Actb
Gapdh
Eef1b2
Eno1b

Fig. S15

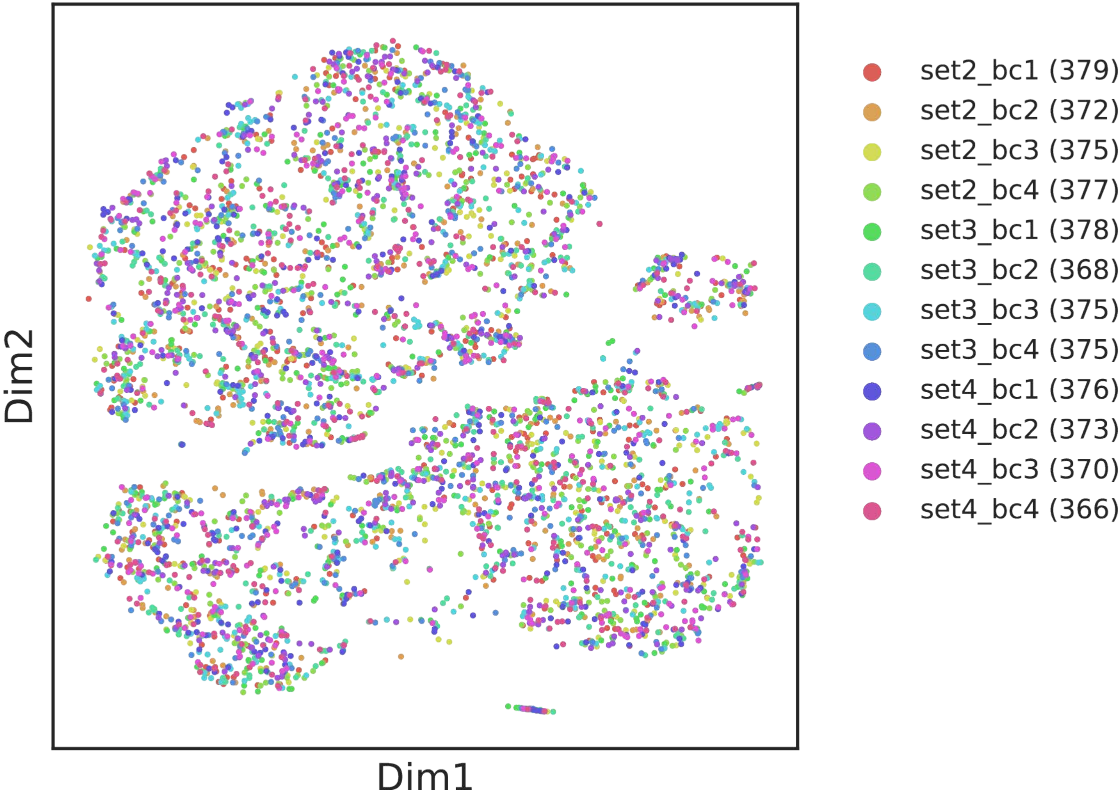
Dim2
Dim1
set2_bc1 (379)
set2_bc2 (372)
set2_bc3 (375)
set2_bc4 (377)
set3_bc1 (378)
set3_bc2 (368)
set3_bc3 (375)
set3_bc4 (375)
set4_bc1 (376)
set4_bc2 (373)
set4_bc3 (370)
set4_bc4 (366)

Fig. S16

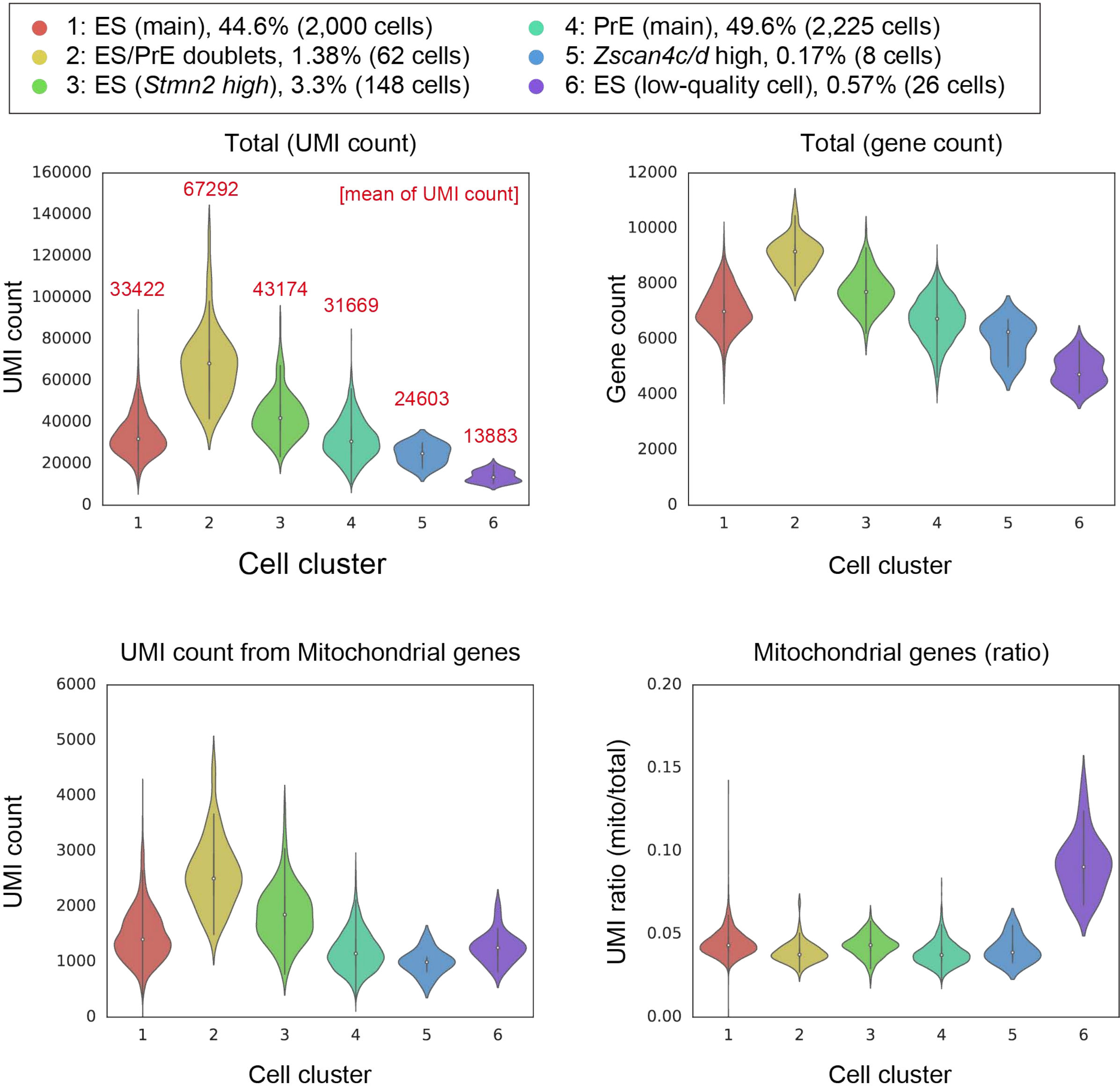
1: ES (main), 44.6% (2,000 cells)
2: ES/PrE doublets, 1.38% (62 cells)
3: ES (*Stmn2 high*), 3.3% (148 cells)
4: PrE (main), 49.6% (2,225 cells)
5: *Zscan4c/d* high, 0.17% (8 cells)
6: ES (low-quality cell), 0.57% (26 cells)
Total (UMI count)
[mean of UMI count]
33422
67292
43174
31669
24603
13883
UMI count
Cell cluster
Total (gene count)
Gene count
Cell cluster
UMI count from Mitochondrial genes
UMI count
Cell cluster
Mitochondrial genes (ratio)
UMI ratio (mito/total)
Cell cluster

Fig. S17

<ESC>

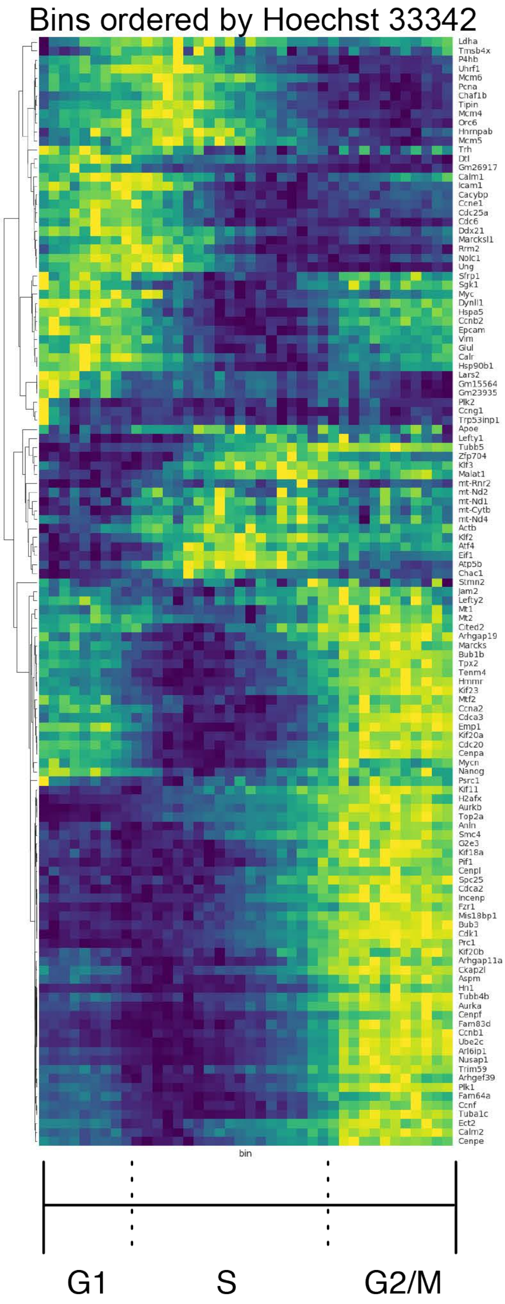

<PrE>

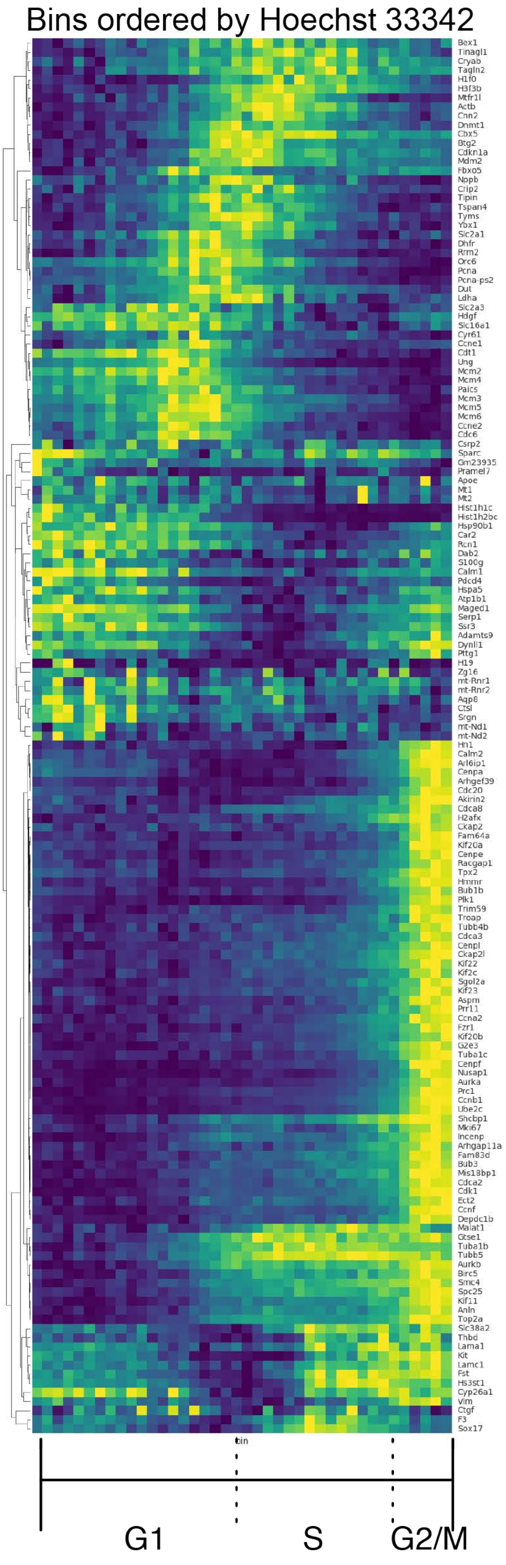

Fig. S18

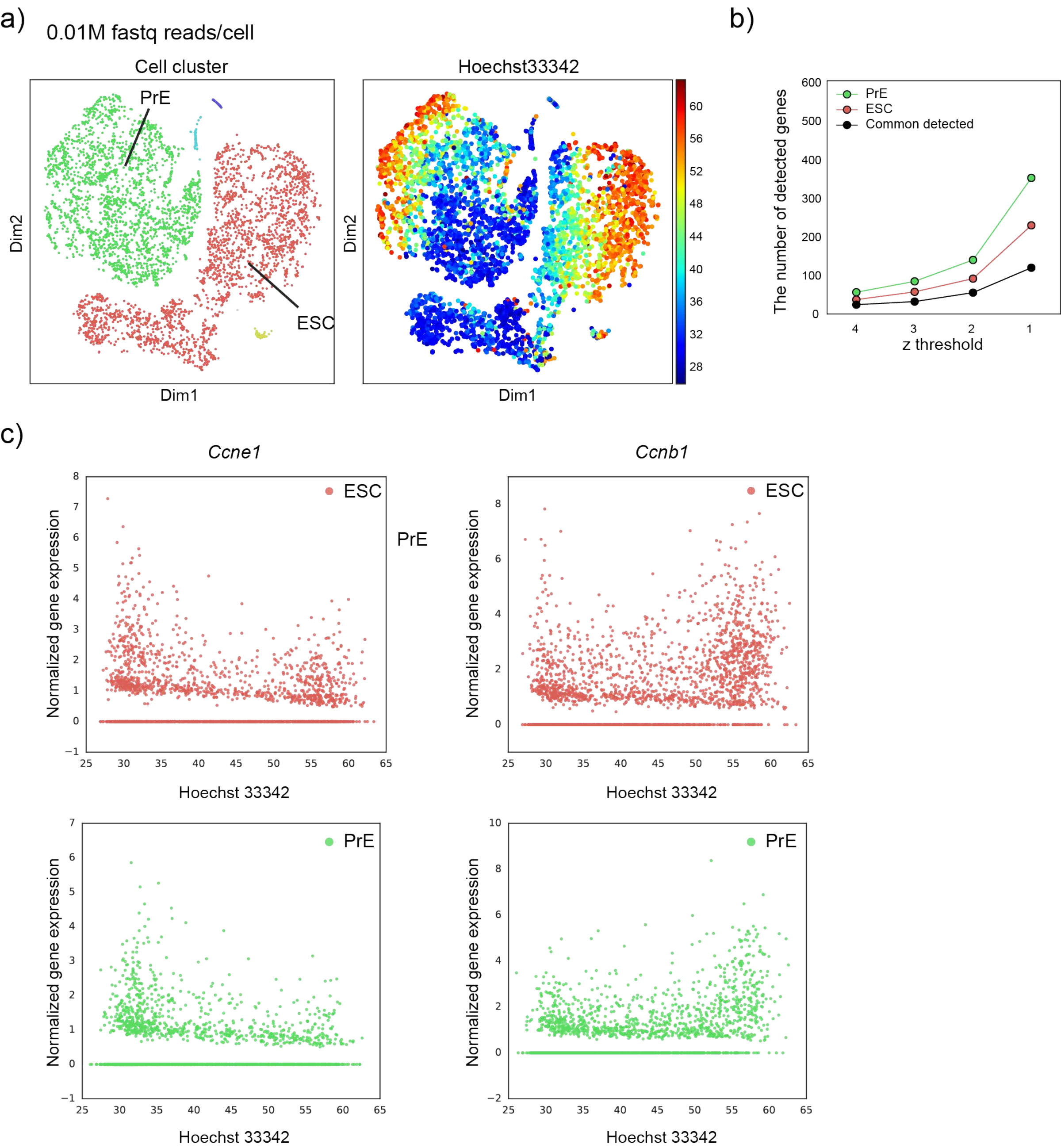
a)
0.01M fastq reads/cell
Cell cluster
PrE
ESC
Dim1
Dim2
Hoechst33342
60
56
52
48
44
40
36
32
28
b)
PrE
ESC
Common detected
The number of detected genes
z threshold
c)
Ccne1
Ccnb1
ESC
PrE
Normalized gene expression
Hoechst 33342

Fig. S19

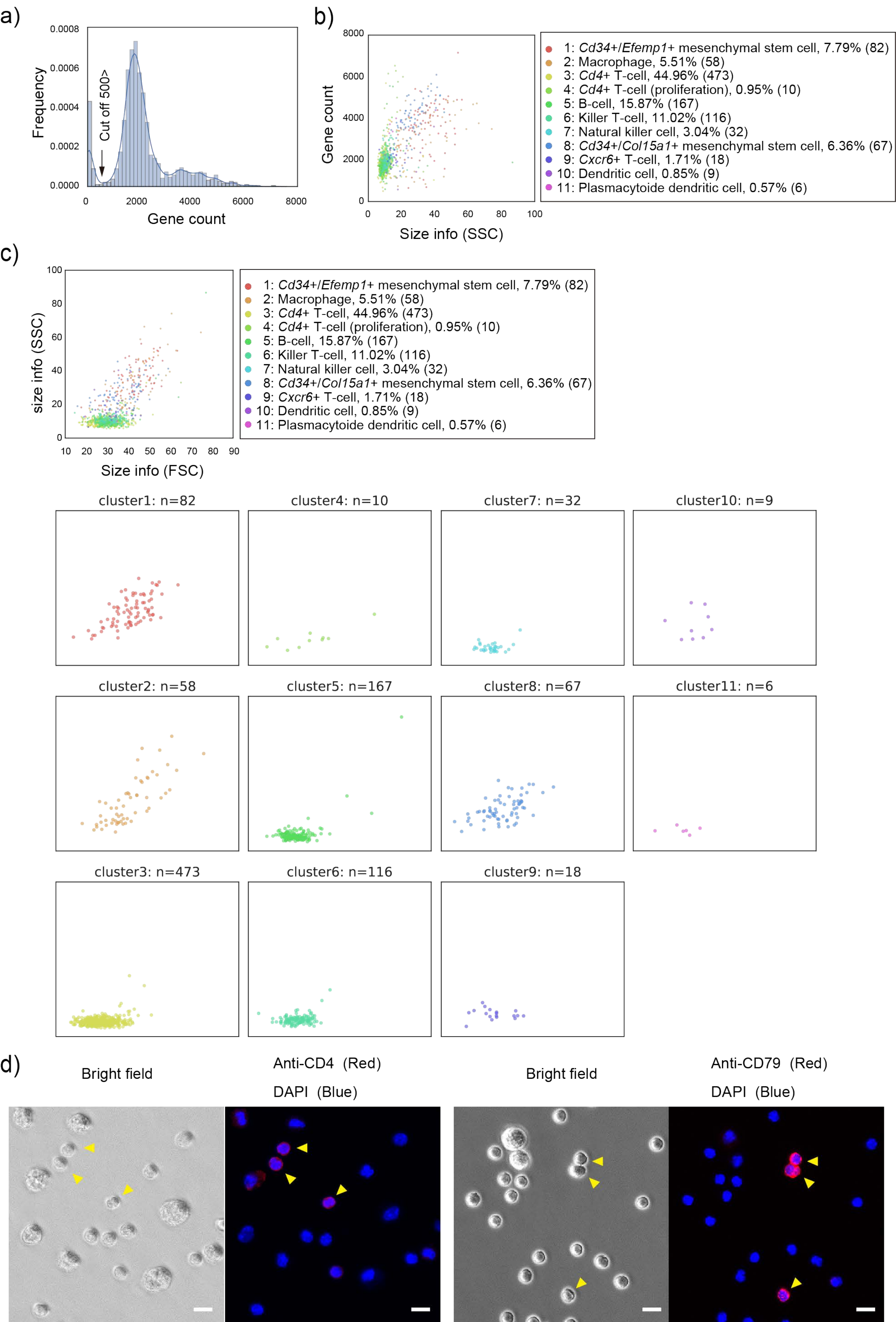
a)
Frequency
Cut off 500>
Gene count
b)
Gene count
Size info (SSC)
1: Cd34+/Efemp1+ mesenchymal stem cell, 7.79% (82)
2: Macrophage, 5.51% (58)
3: Cd4+ T-cell, 44.96% (473)
4: Cd4+ T-cell (proliferation), 0.95% (10)
5: B-cell, 15.87% (167)
6: Killer T-cell, 11.02% (116)
7: Natural killer cell, 3.04% (32)
8: Cd34+/Col15a1+ mesenchymal stem cell, 6.36% (67)
9: Cxcr6+ T-cell, 1.71% (18)
10: Dendritic cell, 0.85% (9)
11: Plasmacytoide dendritic cell, 0.57% (6)
c)
size info (SSC)
Size info (FSC)
cluster1: n=82
cluster4: n=10
cluster7: n=32
cluster10: n=9
cluster2: n=58
cluster5: n=167
cluster8: n=67
cluster11: n=6
cluster3: n=473
cluster6: n=116
cluster9: n=18
d)
Bright field
Anti-CD4 (Red)
DAPI (Blue)
Bright field
Anti-CD79 (Red)
DAPI (Blue)

Fig. S20

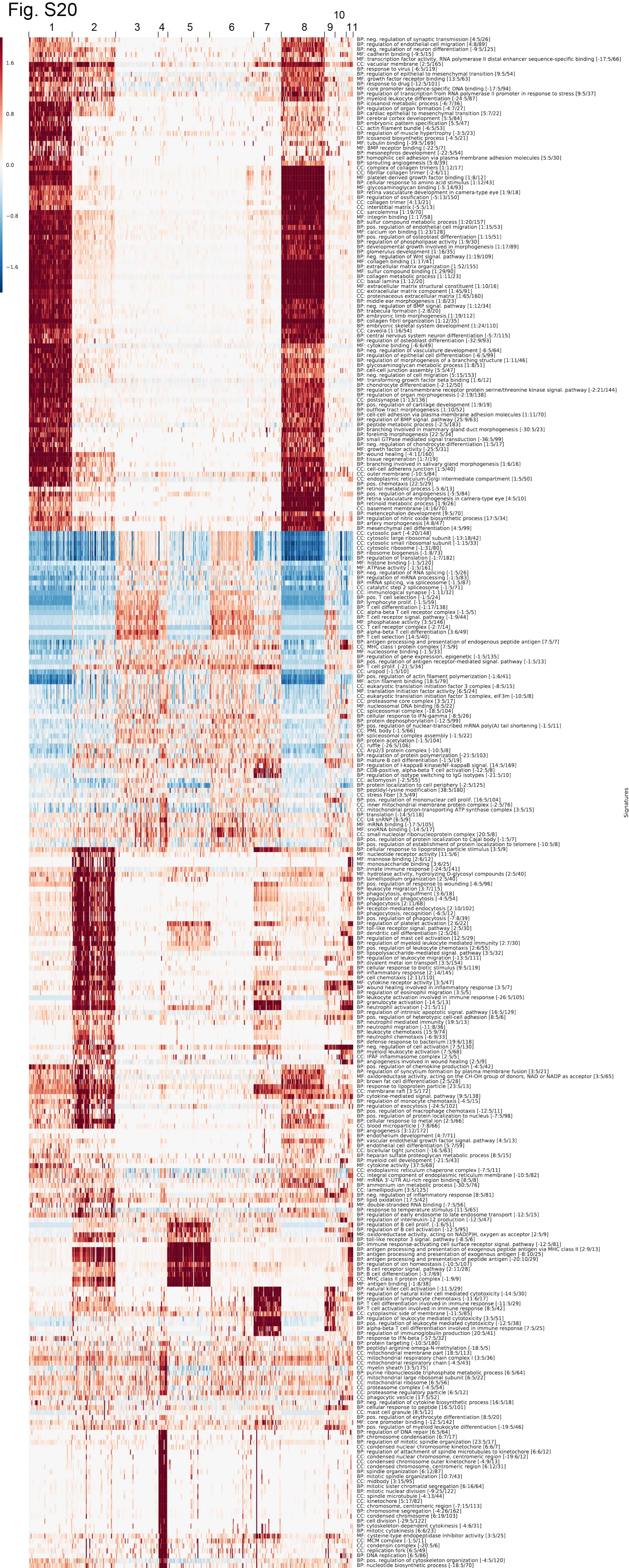
10
1
2
3
4
5
6
7
8
9
11
1.6
0.8
0.0
−0.8
−1.6
Signatures
BP: neg. regulation of synaptic transmission [4:5/26]
BP: regulation of endothelial cell migration [4:8/89]
BP: neg. regulation of neuron differentiation [-9:5/125]
MF: cadherin binding [-9:5/15]
MF: transcription factor activity, RNA polymerase II distal enhancer sequence-specific binding [-17:5/66]
CC: vacuolar membrane [2:5/165]
BP: response to virus [-6:5/119]
BP: regulation of epithelial to mesenchymal transition [9:5/54]
MF: growth factor receptor binding [13:5/63]
BP: response to drug [-12:5/101]
MF: core promoter sequence-specific DNA binding [-17:5/94]
BP: regulation of transcription from RNA polymerase II promoter in response to stress [9:5/37]
BP: myeloid leukocyte differentiation [-24:5/87]
BP: icosanoid metabolic process [-6:7/36]
BP: regulation of organ formation [-4:7/27]
BP: cardiac epithelial to mesenchymal transition [5:7/22]
BP: cerebral cortex development [5:5/84]
BP: embryonic pattern specification [5:5/47]
CC: actin filament bundle [-6:5/53]
BP: regulation of muscle hypertrophy [-3:5/23]
BP: icosanoid biosynthetic process [-4:5/21]
MF: tubulin binding [-39:5/169]
MF: BMP receptor binding [-22:5/7]
BP: mesonephros development [-22:5/54]
BP: homophilic cell adhesion via plasma membrane adhesion molecules [5:5/30]
BP: sprouting angiogenesis [5:8/39]
CC: complex of collagen trimers [1:12/17]
CC: fibrillar collagen trimer [-2:6/11]
MF: platelet-derived growth factor binding [1:8/12]
BP: cellular response to amino acid stimulus [1:12/43]
MF: glycosaminoglycan binding [-5:14/93]
BP: retina vasculature development in camera-type eye [1:9/18]
BP: regulation of ossification [-5:13/150]
CC: collagen trimer [4:13/21]
CC: interstitial matrix [-5:5/13]
CC: sarcolemma [1:19/70]
MF: integrin binding [1:17/58]
BP: sulfur compound metabolic process [1:20/157]
BP: pos. regulation of endothelial cell migration [1:15/53]
MF: calcium ion binding [1:23/128]
BP: pos. regulation of osteoblast differentiation [1:15/51]
BP: regulation of phospholipase activity [1:9/30]
BP: developmental growth involved in morphogenesis [1:17/89]
BP: glomerulus development [1:16/35]
BP: neg. regulation of Wnt signal. pathway [1:19/109]
MF: collagen binding [1:17/41]
BP: extracellular matrix organization [1:52/155]
MF: sulfur compound binding [1:29/90]
BP: collagen metabolic process [1:11/23]
CC: basal lamina [1:12/20]
MF: extracellular matrix structural constituent [1:10/16]
CC: extracellular matrix component [1:45/91]
CC: proteinaceous extracellular matrix [1:65/160]
BP: middle ear morphogenesis [1:8/23]
BP: neg. regulation of BMP signal. pathway [1:12/34]
BP: trabecula formation [-2:8/20]
BP: embryonic limb morphogenesis [1:19/112]
BP: collagen fibril organization [1:12/35]
BP: embryonic skeletal system development [1:24/110]
CC: caveola [1:16/54]
BP: central nervous system neuron differentiation [-5:7/115]
BP: regulation of osteoblast differentiation [-32:9/93]
MF: cytokine binding [-6:6/49]
BP: neg. regulation of vasculature development [-6:5/64]
BP: regulation of epithelial cell differentiation [-6:5/99]
BP: regulation of morphogenesis of a branching structure [1:11/46]
BP: glycosaminoglycan metabolic process [1:8/51]
BP: cell-cell junction assembly [5:5/47]
BP: neg. regulation of cell migration [5:15/153]
MF: transforming growth factor beta binding [1:6/12]
BP: chondrocyte differentiation [-2:12/50]
BP: regulation of transmembrane receptor protein serine/threonine kinase signal. pathway [-2:21/144]
BP: regulation of organ morphogenesis [-2:19/138]
CC: postsynapse [1:13/136]
BP: pos. regulation of cartilage development [1:9/19]
BP: outflow tract morphogenesis [1:10/52]
BP: cell-cell adhesion via plasma-membrane adhesion molecules [1:11/70]
BP: regulation of BMP signal. pathway [25:9/63]
BP: peptide metabolic process [-2:5/183]
BP: branching involved in mammary gland duct morphogenesis [-30:5/23]
BP: forelimb morphogenesis [22:5/34]
BP: small GTPase mediated signal transduction [-36:5/99]
BP: neg. regulation of chondrocyte differentiation [1:5/17]
MF: growth factor activity [-25:5/31]
BP: wound healing [-4:11/160]
BP: tissue regeneration [1:7/19]
BP: branching involved in salivary gland morphogenesis [1:6/16]
CC: cell-cell adherens junction [1:5/40]
CC: outer membrane [-10:5/84]
CC: endoplasmic reticulum-Golgi intermediate compartment [1:5/50]
BP: pos. chemotaxis [22:5/29]
BP: retinol metabolic process [-5:6/13]
BP: pos. regulation of angiogenesis [-5:5/84]
BP: retina vasculature morphogenesis in camera-type eye [4:5/10]
BP: retinoid metabolic process [1:9/26]
CC: basement membrane [4:16/70]
BP: metencephalon development [9:5/70]
BP: regulation of nitric oxide biosynthetic process [17:5/34]
BP: artery morphogenesis [4:8/47]
BP: mesenchymal cell differentiation [4:5/99]
CC: cytosolic part [-4:20/148]
CC: cytosolic large ribosomal subunit [-13:18/42]
CC: cytosolic small ribosomal subunit [-1:15/33]
CC: cytosolic ribosome [-1:31/80]
BP: ribosome biogenesis [-1:8/73]
BP: regulation of translation [-1:7/182]
MF: histone binding [-1:5/120]
MF: ATPase activity [-1:5/161]
BP: neg. regulation of RNA splicing [-1:5/26]
BP: regulation of mRNA processing [-1:5/83]
BP: mRNA splicing, via spliceosome [-1:5/87]
CC: catalytic step 2 spliceosome [-1:5/71]
CC: immunological synapse [-1:11/32]
BP: pos. T cell selection [-1:5/24]
BP: lymphocyte prolif. [-1:5/59]
BP: T cell differentiation [-1:17/138]
CC: alpha-beta T cell receptor complex [-1:5/5]
BP: T cell receptor signal. pathway [-1:9/44]
MF: phosphatase activity [3:5/146]
CC: T cell receptor complex [-2:7/14]
BP: alpha-beta T cell differentiation [3:6/49]
BP: T cell selection [14:5/40]
BP: antigen processing and presentation of endogenous peptide antigen [7:5/7]
CC: MHC class I protein complex [7:5/9]
MF: nucleosome binding [-1:5/33]
BP: regulation of gene expression, epigenetic [-1:5/135]
BP: pos. regulation of antigen receptor-mediated signal. pathway [-1:5/13]
BP: T cell prolif. [-21:5/34]
CC: uropod [-1:5/10]
BP: pos. regulation of actin filament polymerization [-1:6/41]
MF: actin filament binding [18:5/79]
CC: eukaryotic translation initiation factor 3 complex [-8:5/15]
MF: translation initiation factor activity [6:5/24]
CC: eukaryotic translation initiation factor 3 complex, eIF3m [-10:5/8]
CC: proteasome core complex [3:5/17]
MF: nucleosomal DNA binding [6:5/22]
CC: spliceosomal complex [-18:5/104]
BP: cellular response to IFN-gamma [-8:5/26]
BP: protein dephosphorylation [-12:5/99]
BP: pos. regulation of nuclear-transcribed mRNA poly(A) tail shortening [-1:5/11]
CC: PML body [-1:5/66]
BP: spliceosomal complex assembly [-1:5/22]
BP: protein acetylation [-1:5/104]
CC: ruffle [-26:5/106]
CC: Arp2/3 protein complex [-10:5/8]
BP: regulation of protein polymerization [-21:5/103]
BP: mature B cell differentiation [-1:5/19]
BP: regulation of I-kappaB kinase/NF-kappaB signal. [14:5/169]
BP: CD8-positive, alpha-beta T cell activation [-12:5/8]
BP: regulation of isotype switching to IgG isotypes [-21:5/10]
CC: actomyosin [-2:5/55]
BP: protein localization to cell periphery [-2:5/125]
BP: peptidyl-lysine modification [38:5/180]
CC: stress fiber [3:5/49]
BP: pos. regulation of mononuclear cell prolif. [16:5/104]
CC: inner mitochondrial membrane protein complex [-2:5/76]
CC: mitochondrial proton-transporting ATP synthase complex [3:5/15]
BP: translation [-14:5/118]
CC: U4 snRNP [6:5/9]
MF: mRNA binding [-17:5/105]
MF: snoRNA binding [-14:5/17]
CC: small nucleolar ribonucleoprotein complex [20:5/8]
BP: pos. regulation of protein localization to Cajal body [-1:5/7]
BP: pos. regulation of establishment of protein localization to telomere [-10:5/8]
BP: cellular response to lipoprotein particle stimulus [3:5/9]
MF: nucleotide receptor activity [11:5/6]
MF: mannose binding [2:6/12]
MF: monosaccharide binding [3:6/25]
BP: innate immune response [-24:5/141]
MF: hydrolase activity, hydrolyzing O-glycosyl compounds [2:5/40]
BP: lamellipodium organization [2:5/40]
BP: pos. regulation of response to wounding [-6:5/96]
BP: leukocyte migration [3:7/115]
BP: phagocytosis, engulfment [3:6/18]
BP: regulation of phagocytosis [-4:5/54]
BP: phagocytosis [2:11/68]
BP: receptor-mediated endocytosis [2:10/102]
BP: phagocytosis, recognition [-6:5/12]
BP: pos. regulation of phagocytosis [-7:8/39]
BP: regulation of platelet activation [2:6/22]
BP: toll-like receptor signal. pathway [2:5/30]
BP: dendritic cell differentiation [2:5/26]
BP: regulation of mast cell activation [12:5/29]
BP: regulation of myeloid leukocyte mediated immunity [2:7/30]
BP: pos. regulation of leukocyte chemotaxis [2:6/55]
BP: lipopolysaccharide-mediated signal. pathway [3:5/32]
BP: regulation of leukocyte migration [-13:5/111]
BP: divalent metal ion transport [3:5/154]
BP: cellular response to biotic stimulus [9:5/119]
BP: inflammatory response [2:14/145]
BP: cell chemotaxis [2:11/110]
MF: cytokine receptor activity [3:5/47]
BP: wound healing involved in inflammatory response [3:5/7]
BP: regulation of eosinophil migration [3:5/5]
BP: leukocyte activation involved in immune response [-26:5/105]
BP: granulocyte activation [-14:5/13]
BP: neutrophil activation [-21:5/11]
BP: regulation of intrinsic apoptotic signal. pathway [16:5/129]
BP: pos. regulation of heterotypic cell-cell adhesion [8:5/6]
BP: neutrophil mediated immunity [19:5/13]
BP: neutrophil migration [-11:8/36]
BP: leukocyte chemotaxis [15:9/74]
BP: neutrophil chemotaxis [-6:9/33]
BP: defense response to bacterium [19:6/118]
BP: neg. regulation of cell activation [7:5/130]
BP: myeloid leukocyte activation [7:5/68]
CC: IPAF inflammasome complex [2:5/5]
BP: angiogenesis involved in wound healing [2:5/9]
BP: pos. regulation of chemokine production [-4:5/42]
BP: regulation of syncytium formation by plasma membrane fusion [3:5/21]
MF: oxidoreductase activity, acting on the CH-OH group of donors, NAD or NADP as acceptor [3:5/65]
BP: brown fat cell differentiation [-2:5/28]
BP: response to lipoprotein particle [23:5/13]
CC: membrane raft [3:5/172]
BP: cytokine-mediated signal. pathway [9:5/138]
BP: regulation of monocyte chemotaxis [-4:5/15]
BP: regulation of exocytosis [-24:5/102]
BP: pos. regulation of macrophage chemotaxis [-12:5/11]
BP: pos. regulation of protein localization to nucleus [-7:5/98]
BP: cellular response to metal ion [2:5/66]
CC: blood microparticle [-7:8/66]
BP: angiogenesis [3:12/172]
BP: endothelium development [4:7/71]
BP: vascular endothelial growth factor signal. pathway [4:5/13]
BP: endothelial cell differentiation [5:7/59]
CC: bicellular tight junction [-16:5/63]
BP: heparan sulfate proteoglycan metabolic process [8:5/15]
BP: myeloid cell development [-21:5/43]
MF: cytokine activity [37:5/68]
CC: endoplasmic reticulum chaperone complex [-7:5/11]
CC: integral component of endoplasmic reticulum membrane [-10:5/82]
MF: mRNA 3'-UTR AU-rich region binding [8:5/8]
BP: ammonium ion metabolic process [-30:5/76]
CC: lamellipodium [3:5/125]
BP: neg. regulation of inflammatory response [8:5/81]
BP: lipid oxidation [17:5/42]
MF: double-stranded RNA binding [-7:5/56]
BP: response to temperature stimulus [11:5/65]
BP: regulation of early endosome to late endosome transport [-12:5/15]
BP: regulation of interleukin-12 production [-12:5/47]
BP: regulation of B cell prolif. [-1:6/51]
BP: regulation of B cell activation [-12:5/95]
MF: oxidoreductase activity, acting on NAD(P)H, oxygen as acceptor [2:5/9]
BP: toll-like receptor 3 signal. pathway [-8:5/6]
BP: immune response-activating cell surface receptor signal. pathway [-12:5/81]
BP: antigen processing and presentation of exogenous peptide antigen via MHC class II [2:9/13]
BP: antigen processing and presentation of exogenous antigen [-8:10/25]
BP: antigen processing and presentation of peptide antigen [-20:10/29]
BP: regulation of ion homeostasis [-10:5/107]
BP: B cell receptor signal. pathway [2:11/28]
BP: B cell differentiation [-3:7/69]
CC: MHC class II protein complex [-1:9/9]
MF: antigen binding [-1:8/38]
BP: natural killer cell activation [-11:5/29]
BP: regulation of natural killer cell mediated cytotoxicity [-14:5/30]
BP: regulation of lymphocyte chemotaxis [-11:6/17]
BP: T cell differentiation involved in immune response [-11:5/29]
BP: T cell activation involved in immune response [8:5/42]
CC: cytoplasmic side of membrane [-11:8/85]
BP: regulation of leukocyte mediated cytotoxicity [3:5/51]
BP: pos. regulation of leukocyte mediated cytotoxicity [-12:5/38]
BP: alpha-beta T cell differentiation involved in immune response [7:5/25]
BP: regulation of immunoglobulin production [20:5/41]
BP: response to IFN-beta [-57:5/32]
BP: protein targeting [-10:5/180]
BP: peptidyl-arginine omega-N-methylation [-18:5/5]
CC: mitochondrial membrane part [18:5/113]
CC: mitochondrial respiratory chain complex I [3:5/36]
CC: mitochondrial respiratory chain [-4:5/43]
CC: myelin sheath [3:5/175]
BP: purine ribonucleoside triphosphate metabolic process [6:5/64]
CC: mitochondrial large ribosomal subunit [6:5/22]
CC: mitochondrial ribosome [6:5/56]
CC: proteasome complex [-4:5/54]
CC: proteasome regulatory particle [6:5/12]
CC: phagocytic vesicle [17:5/52]
BP: neg. regulation of cytokine biosynthetic process [16:5/18]
BP: cellular response to peptide [16:5/101]
CC: mast cell granule [8:5/12]
BP: pos. regulation of erythrocyte differentiation [8:5/20]
MF: core promoter binding [-12:5/142]
BP: pos. regulation of myeloid leukocyte differentiation [-19:5/46]
BP: regulation of DNA repair [6:5/64]
BP: chromosome condensation [6:7/17]
BP: regulation of mitotic spindle organization [23:5/17]
CC: condensed nuclear chromosome kinetochore [6:6/7]
BP: regulation of attachment of spindle microtubules to kinetochore [6:6/12]
CC: condensed nuclear chromosome, centromeric region [-19:6/12]
CC: condensed chromosome outer kinetochore [-4:9/13]
CC: condensed chromosome, centromeric region [6:12/31]
BP: spindle organization [6:12/87]
BP: mitotic spindle organization [10:7/43]
CC: midbody [3:15/95]
BP: mitotic sister chromatid segregation [6:16/64]
BP: mitotic nuclear division [-9:25/122]
CC: spindle microtubule [-4:13/44]
CC: kinetochore [5:17/82]
CC: chromosome, centromeric region [-7:15/113]
BP: chromosome segregation [-4:26/162]
CC: condensed chromosome [6:19/103]
BP: cell division [-29:5/122]
BP: cytoskeleton-dependent cytokinesis [-4:6/31]
BP: mitotic cytokinesis [6:6/23]
MF: cysteine-type endopeptidase inhibitor activity [3:5/25]
CC: MCM complex [-1:5/11]
CC: condensin complex [-20:5/6]
CC: replication fork [6:5/49]
BP: DNA replication [6:5/86]
BP: pos. regulation of cytoskeleton organization [-4:5/120]
BP: nucleotide biosynthetic process [-18:5/70]

Fig. S21

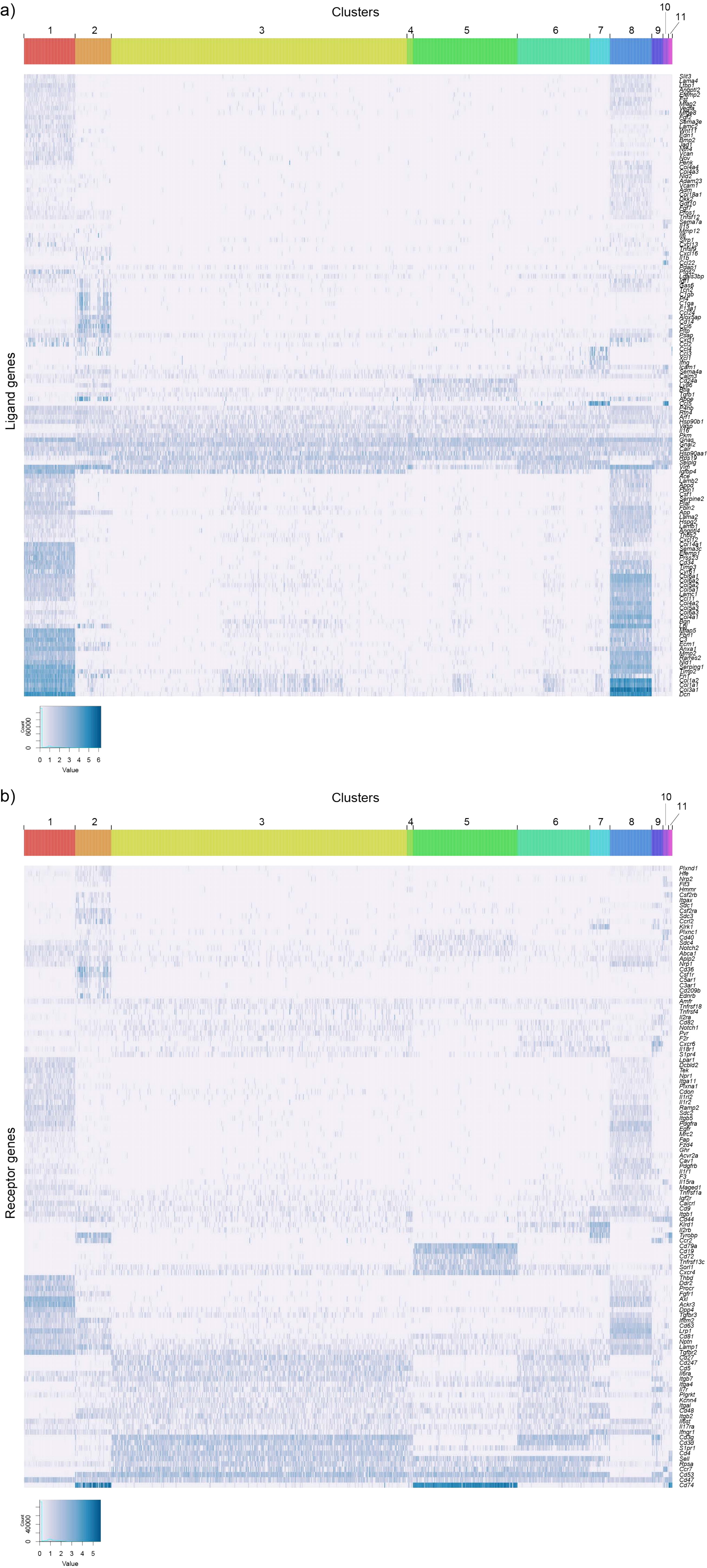

a)
Clusters
1
2
3
4
5
6
7
8
9
10
11
Ligand genes
Count
0
60000
0 1 2 3 4 5 6
Value
b)
Clusters
1
2
3
4
5
6
7
8
9
10
11
Receptor genes
Count
0
40000
0 1 2 3 4 5
Value

Fig. S22

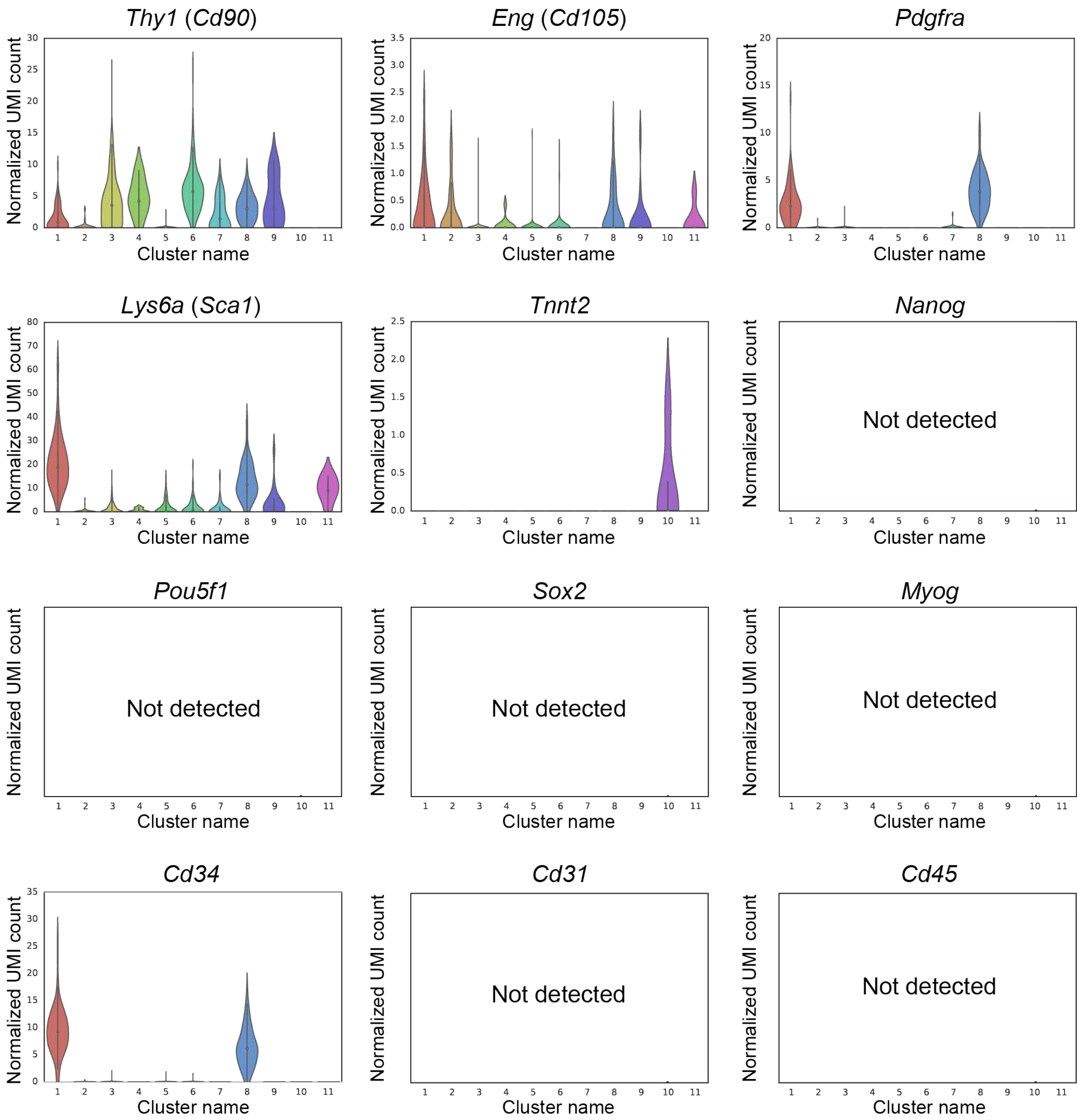

Thy1 (Cd90)
Eng (Cd105)
Pdgfra
Lys6a (Sca1)
Tnnt2
Nanog
Not detected
Pou5f1
Not detected
Sox2
Not detected
Myog
Not detected
Cd34
Cd31
Not detected
Cd45
Not detected
Normalized UMI count
Cluster name

Fig. S23

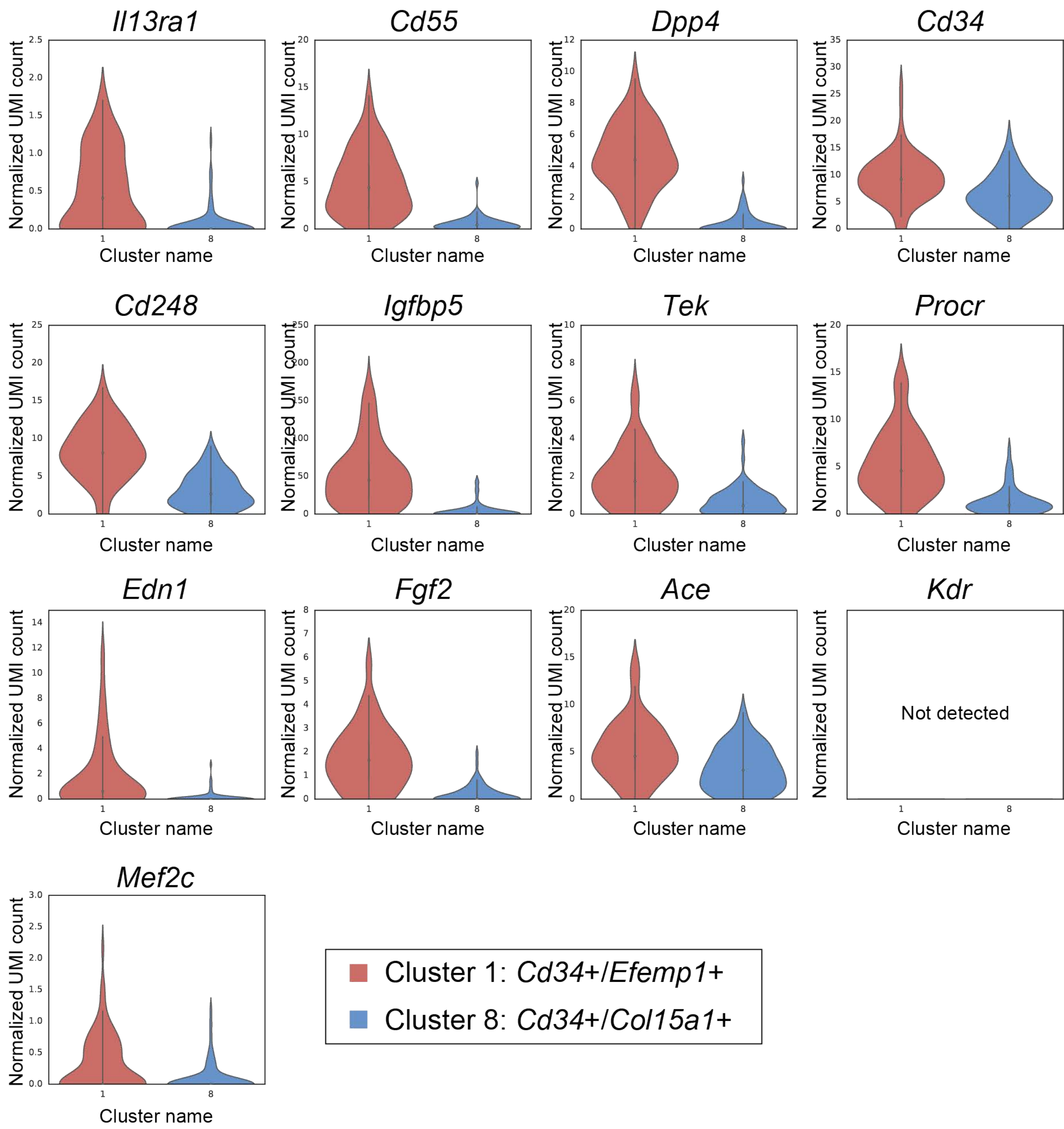

Il13ra1
Cd55
Dpp4
Cd34
Cd248
Igfbp5
Tek
Procr
Edn1
Fgf2
Ace
Kdr
Not detected
Mef2c
Normalized UMI count
Cluster name
Cluster 1: Cd34+/Efemp1+
Cluster 8: Cd34+/Col15a1+

Fig. S24

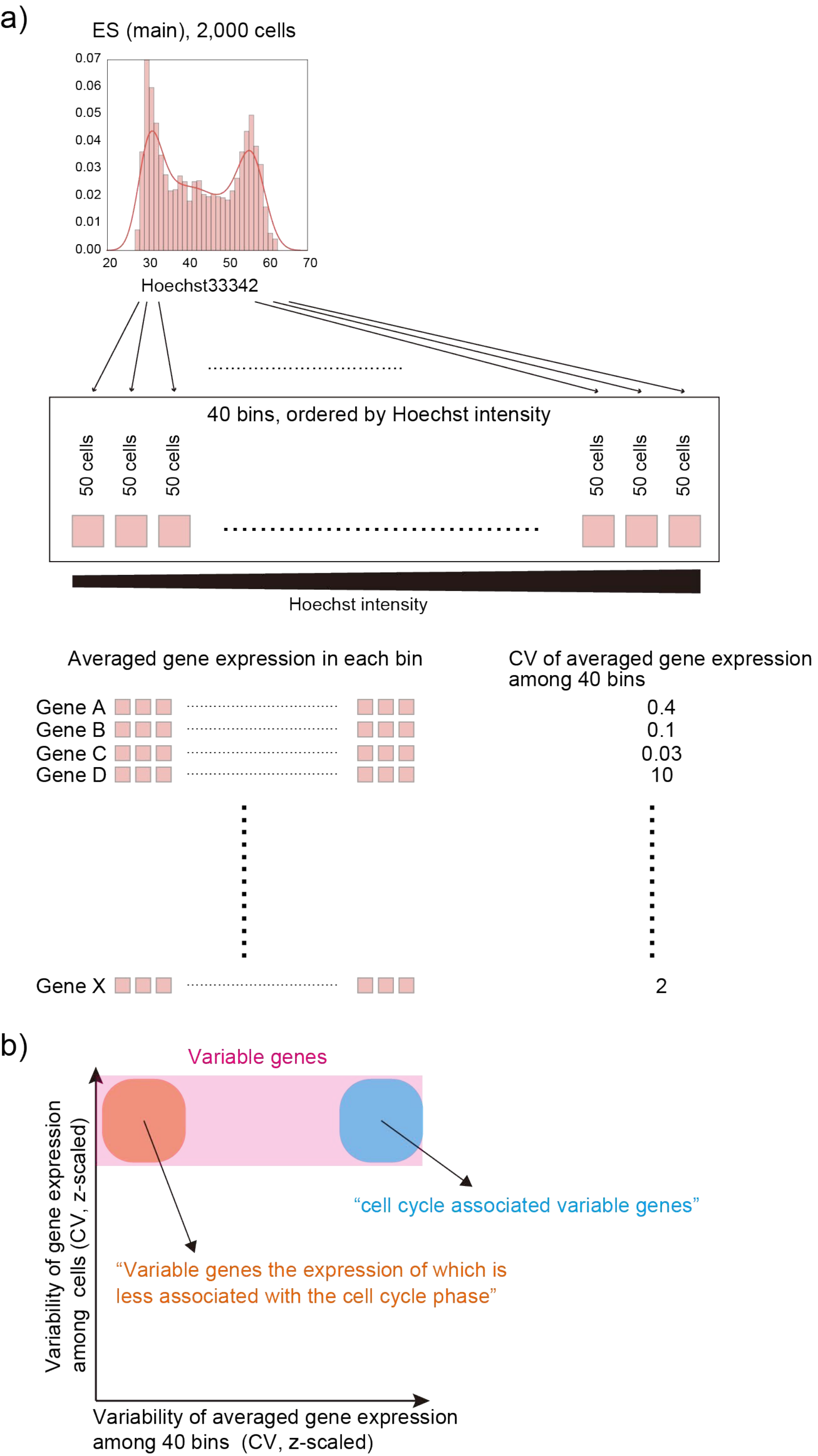
a)
ES (main), 2,000 cells
0.07
0.06
0.05
0.04
0.03
0.02
0.01
0.00
20
30
40
50
60
70
Hoechst33342
40 bins, ordered by Hoechst intensity
50 cells
50 cells
50 cells
50 cells
50 cells
50 cells
Hoechst intensity
Averaged gene expression in each bin
CV of averaged gene expression among 40 bins
Gene A
Gene B
Gene C
Gene D
0.4
0.1
0.03
10
Gene X
2
b)
Variable genes
"cell cycle associated variable genes"
"Variable genes the expression of which is less associated with the cell cycle phase"
Variability of gene expression among cells (CV, z-scaled)
Variability of averaged gene expression among 40 bins (CV, z-scaled)
